# White Rot Fungi Produce Novel Tire Wear Compound Metabolites and Reveal Underappreciated Amino Acid Conjugation Pathways

**DOI:** 10.1021/acs.estlett.2c00114

**Published:** 2022-03-18

**Authors:** Erica A. Wiener, Gregory H. LeFevre

**Affiliations:** † Department of Civil & Environmental Engineering, University of Iowa, 4105 Seamans Center, Iowa City, Iowa 52242, United States; ‡ C. Maxwell Stanley Hydraulics Laboratory, IIHR−Hydroscience & Engineering, Iowa City, Iowa 52242, United States

**Keywords:** tire additives, HMMM, acetanilide, glutamine conjugation, fungal biotransformation, green stormwater infrastructure, *Trametes versicolor*

## Abstract

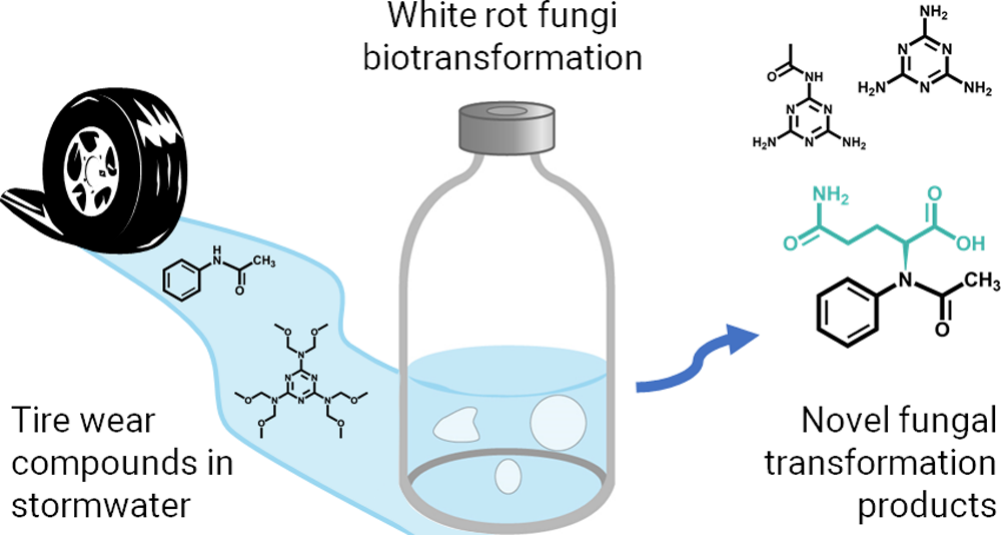

There is increasing concern about tire wear compounds (TWCs) in surface water and stormwater as evidence grows on their toxicity and widespread detection in the environment. Because TWCs are prevalent in stormwater, there is a need to understand fate and treatment options including biotransformation in green infrastructure (e.g., bioretention). Particularly, fungal biotransformation is not well-studied in a stormwater context despite the known ability of certain fungi to remove recalcitrant contaminants. Here, we report the first study on fungal biotransformation of the TWCs acetanilide and hexamethoxymethylmelamine (HMMM). We found that the model white rot fungus, *Trametes versicolor*, removed 81.9% and 69.6% of acetanilide and HMMM, respectively, with no significant sorption to biomass. The bicyclic amine 1,3-diphenylguanidine was not removed. Additionally, we identified novel TWC metabolites using semi-untargeted metabolomics via high-resolution mass spectrometry. Key metabolites include multiple isomers of HMMM biotransformation products, melamine as a possible “dead-end” product of HMMM (verified with an authentic standard), and a glutamine-conjugated product of acetanilide. These metabolites have implications for environmental toxicity and treatment. Our discovery of the first fungal glutamine-conjugated product highlights the need to investigate amino acid conjugation as an important pathway in biotransformation of contaminants, with implications in other fields including natural products discovery.

## Introduction

Tire wear compounds (TWCs) are an emerging class of contaminants increasingly detected in rivers, estuaries, stormwater, and aquatic organisms.^1−4^ Tire wear occurs through mechanical abrasion of tires with roads, and estimated emissions range from 10^6^ to 10^8^ kg/y in different countries.^5−7^ Although empirical data for tire/road wear particles (TRWPs) in the environment are limited, recent studies indicate that TRWPs are abundant microplastics and can comprise the majority of anthropogenic particles in urban stormwater.^8−10^ Once in the environment, compounds within TRWPs can leach into sediments, surface water, and stormwater. These TWCs can be toxic to aquatic biota. For example, the TWC transformation product *N*-phenyl-*N*′-(1,3-dimethylbutyl)-*p*-phenylenediamine (6-PPD) quinone causes acute toxicity in coho salmon, rainbow trout, and brook trout,^11,12^ highlighting the importance of leached TWC toxicity.^13−19^

Multiple TWCs have been detected in stormwater, surface water, or aquatic biota including acetanilide, bicyclic amines, methoxymethylmelamines, and *N*,*N*′-disubstituted phenylenediamines.^20−22^ Acetanilide is used in rubber vulcanization and was detected in road stormwater runoff and fish tissues of runoff-exposed coho salmon.^23^ Compounds within the bicyclic amine class (e.g., 1,3-diphenylguanidine) were among the top three largest peak features in untargeted mass spectrometry analysis of road runoff acutely toxic to coho salmon.^1^ Hexamethoxymethylmelamine (HMMM) was first detected in surface waters in 2002 and has subsequently been measured in surface waters and stormwater globally.^3,20,24−28^ Transformation of HMMM in wastewater results in more polar, mobile products that likely persist in environmental waters, as evidenced by transformation products present in raw drinking waters even after riverbank filtration.^26^ A study proposed 38 abiotic oxidation products of 6-PPD, including highly polar transformation products of 6-PPD in snow, demonstrating the complex distribution of TWC in the environment.^29^ The extent of environmental loading and growing concern about TWC toxicity warrant timely scrutiny, especially concerning environmental fate, transformation, and treatment options.

Novel or enhanced treatment strategies are urgently needed to address TWCs in stormwater. Green infrastructure such as bioretention cells can remove contaminants and reduce aquatic toxicity through both abiotic and biotic processes.^30−32^ Following initial abiotic sorption to soil, mulch, or amendments such as biochar,^33−35^ contaminants could be biologically transformed, thereby restoring sorption capacity. Nevertheless, studies on biotransformation in bioretention are often limited to bacteria or plants, with a paucity on fungi. To date, research on fungi in bioretention has largely focused on mycorrhizal fungi, or fungi that form mutualistic relationships with plants.^36−38^ Beyond mycorrhizal fungi, white rot fungi could be leveraged to promote biotic treatment.^39^ It is well-established that white rot fungi can grow in diverse environments^40−43^ and can use extracellular enzymes to transform lignin, polycyclic aromatic hydrocarbons, and other recalcitrant compounds.^44−47^ To better understand the potential role of fungi in treating TWCs in green infrastructure, we investigated the removal of known TWCs by the model white rot fungus, *Trametes versicolor*. Our primary research objectives were to quantify *T. versicolor* removal of the TWCs acetanilide, 1,3-diphenylguanidine, and hexamethoxymethylmelamine and to identify novel TWC fungal metabolites. This is the first study to demonstrate fungal removal of any TWCs and provides insight into the environmental fate and possible treatment of TWCs in stormwater.

## Materials and Methods

### Chemicals

All chemical information can be found in Section S1. Synthetic stormwater containing major ions commonly found in urban stormwater^34,39,48^ was created at 10× concentration, diluted with deionized water, and pH-adjusted to 7.0 ± 0.2 prior to autoclaving.

### Fungal Cultures

Before each batch experiment, liquid cultures were grown by inoculating *Trametes versicolor* (ATCC 42530) into malt extract media and incubating on a shaker at room temperature for 5–7 days (see Section S2 for further details on fungal maintenance and culture conditions).

### Experimental Design

#### Kinetics of TWC Fungal Removal

Matched-pairs batch experiments were conducted using abiotic controls and fungal treatments to measure fungal removal of HMMM and acetanilide in synthetic stormwater. Abiotic controls contained synthetic stormwater and the target analyte. Fungal treatments contained synthetic stormwater, the target analyte, and equal volume aliquots of homogenized *T. versicolor*. The experiments were conducted in triplicate with sacrificial sampling on days 0, 3, 8, and 15. To test sorption of HMMM to fungal biomass, a separate experiment was conducted with fungal treatments and a fungal sorption control. The fungal sorption controls were treated with sodium azide and sacrificially sampled. See Section S3 for further details.

#### Sampling for Metabolites from Fungal Biomass and Extracellular Samples

Six biological replicates each were prepared for TWC-exposed and unexposed fungi. Unexposed fungi controls contained synthetic stormwater and homogenized *T. versicolor*. Biomass and extracellular samples were collected at days 7 and 15 (samples collected using sterile loops for biomass, syringes for extracellular samples). Metabolites were extracted from biomass using adapted, previously published methods^39^ (see Section S4).

#### Analytical Methods

TWC concentrations were quantified using an Agilent 1260 liquid chromatography system with diode array detection (DAD). Method development and chromatography details are in Section S5. All statistical analyses were performed in GraphPad Prism 9.0.0 (San Diego, CA). To determine if concentration significantly changed over time, we tested departure from the null slope (α = 0.05). Kinetics rate constants were calculated by generating zero-, first-, and second-order equation fits for the data.

For fungal metabolite detection of HMMM and acetanilide, we quantitatively compared metabolite profiles of TWC-exposed and unexposed fungi. We measured metabolites in both extracellular media and biomass-extracted samples. A minimum of three randomly selected, time-matched samples were analyzed on a Thermo Fisher Q Exactive high-resolution Orbitrap mass spectrometer at the University of Iowa High Resolution Mass Spectrometry (HRMS) Facility. Samples were run with polarity switching (positive and negative modes), and composite samples with equal aliquots of biological replicates were used for ddMS^2^. Details on ionization modes, chromatography method, and instrument settings are in Section S5. HRMS data analysis, including differential analysis between TWC-exposed and unexposed fungi, was conducted using Compound Discoverer (version 3.1) and Thermo Fisher Freestyle (1.8.51.0). See Section S6 for Compound Discoverer workflow and modifications. To communicate confidence in compound identification, we used the Schymanski framework.^49^

## Results and Discussion

### Kinetics

**Figure 1:**
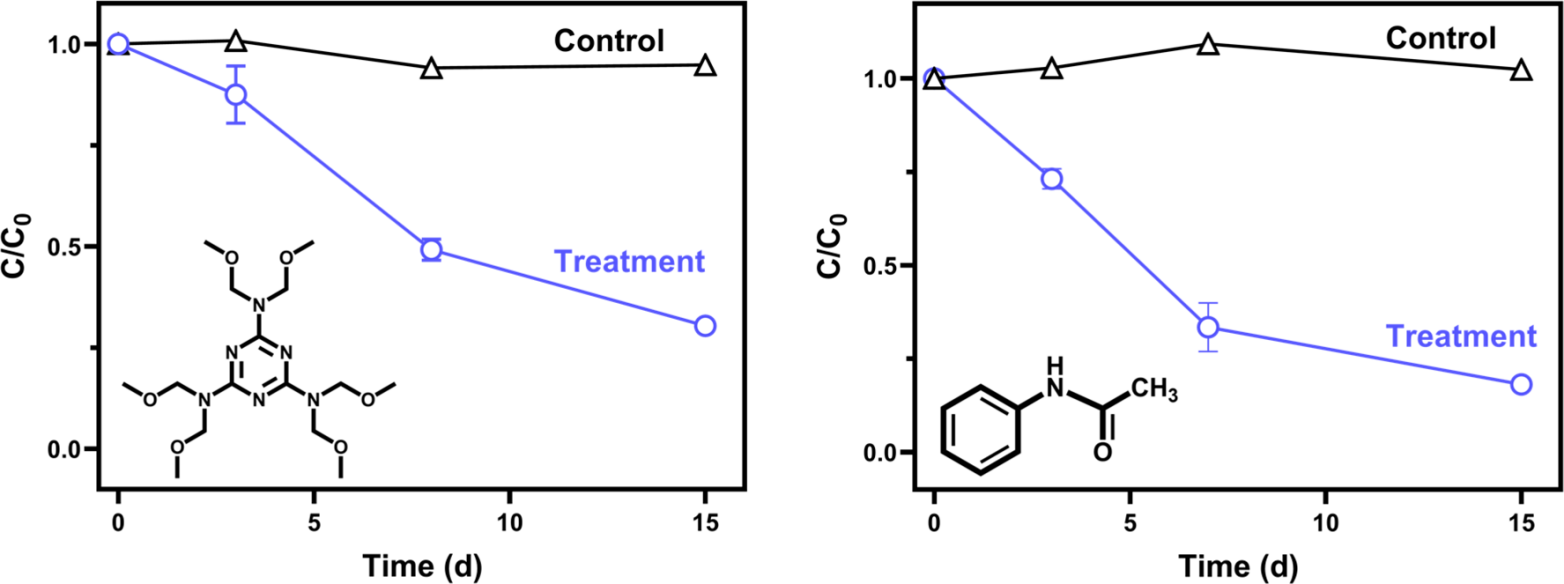
Relative concentration of HMMM (left) and acetanilide (right) through time for abiotic controls (black △) and fungal treatments (blue ○). Error bars represent standard error about the mean for triplicate samples. Some error bars are small and obscured by the data symbols. There was no significant loss of acetanilide (*p* = 0.5177) or HMMM (*p* = 0.9559) in the abiotic controls. For acetanilide, the first-order removal rate was [*C*_0_ = 21.7 μM; *k* ± standard error = 0.116 ± 0.015 day^–1^]. For HMMM, the first-order removal rate was [*C*_0_ = 12.6 μM; *k* ± standard error = 0.087 ± 0.008 day^–1^]. A comparison of kinetics models and absolute measured concentrations are available in Section S7.

Over 15 days, the presence of the white rot fungus *Trametes versicolor* decreased concentrations of the TWCs acetanilide and HMMM in synthetic stormwater by 81.9 ± 0.8% and 69.6 ± 0.5%, respectively (Figure 1). The first-order rate constants were *k* ± standard error = 0.116 ± 0.015 day^–1^ for acetanilide and 0.087 ± 0.008 day^–1^ for HMMM. Sorption to biomass was not significant over 15 days for HMMM (*p* = 0.1351, Figure S1). Given HMMM’s higher log *K*_ow_ relative to acetanilide^50^ (acetanilide log *K*_ow_ = 1.16; HMMM predicted log *K*_ow_ = 1.61, EPISUITE), sorption to biomass was only tested for HMMM. Altogether, the high loss and lack of sorption implicate fungal biotransformation as the primary removal mechanism of acetanilide and HMMM. In contrast, 1,3-diphenylguanidine was not significantly removed (*p* > 0.05; Figure S2). We currently do not know why 1,3-diphenylguanidine was not removed; *T. versicolor* produces enzymes capable of degrading diverse anthropogenic compounds.^46,51,52^ It is possible that *T. versicolor* could degrade 1,3-diphenylguanidine under extended time periods or different growth conditions. Growth conditions, including culturing with solid surfaces/substrates, can impact contaminant removal and should be investigated in future studies.^53,54^

### Established and Novel HMMM Transformation Products

**Figure 2:**
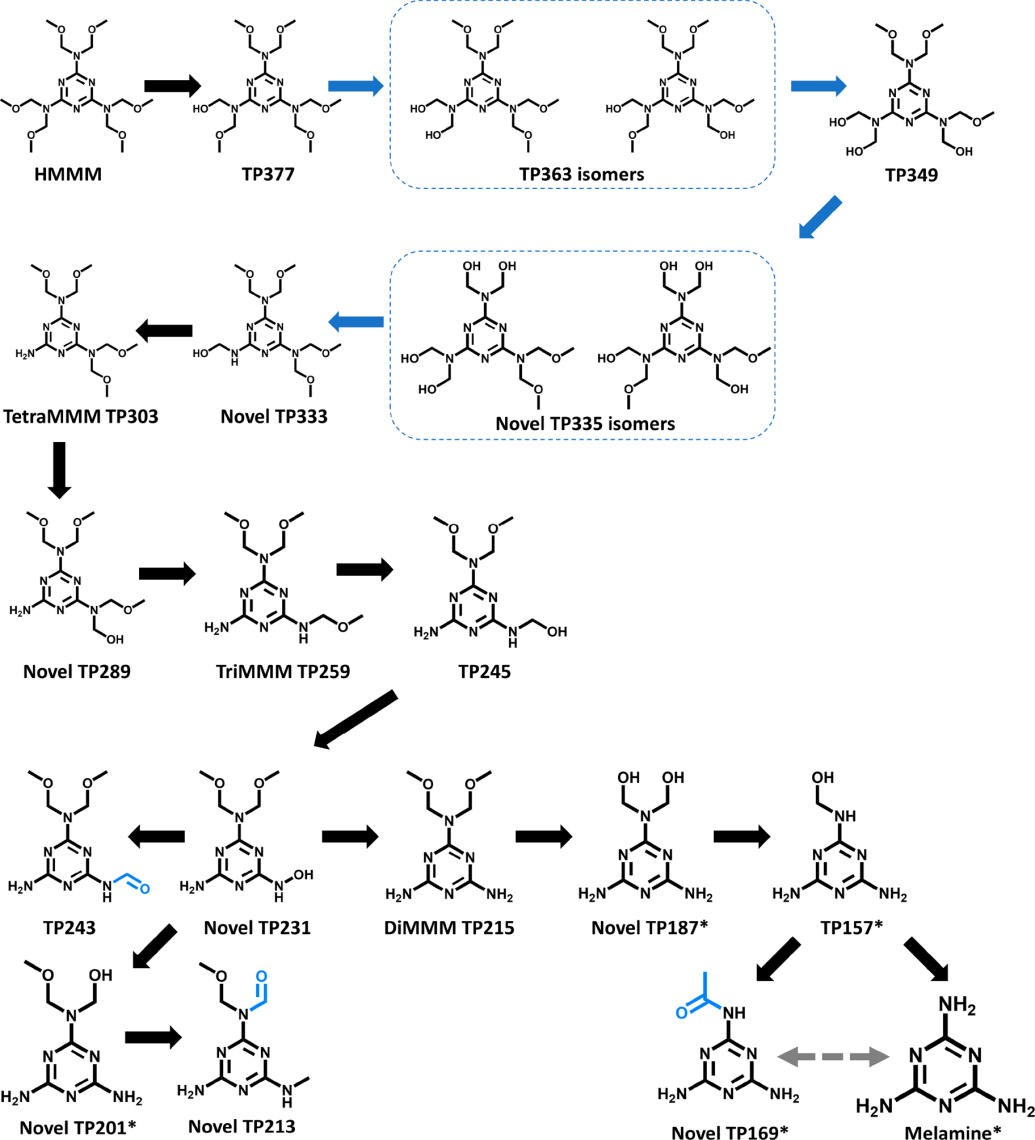
Proposed *T. versicolor* transformation pathway for HMMM with previously undiscovered products labeled as “Novel”. Precise determination of which branch the breakdown occurs is not possible with our methods due to the symmetry of the molecule. There are 20 transformation products in this pathway; of these, 10 are previously unreported. Confidence levels (Schymanski framework) and annotated MS^2^ fragmentation patterns for transformation products are detailed in Sections S8 and S9. Melamine was confirmed with a reference standard. TP377 is likely transformed to TP363 and an isomer because we observed two compounds with the same [M + H]^+^*m*/*z* = 363.1603 and similar fragmentation but at two distinct retention times (26.89 and 27.49 min). The exact positions of the hydroxyl groups cannot be determined on the isomer; thus, a proposed example is depicted, and the two TP363 are highlighted with a dashed blue box. Similarly, the novel TP335 had a structural isomer. Aldehyde and carboxylate groups are also highlighted in blue. The gray dashed arrow indicates the possibility of transformation of TP169 to melamine (i.e., decarboxylation) and melamine to TP169 via acetylation. Compounds marked with an asterisk (*) could only be identified in extracellular samples.

We discovered multiple novel HMMM fungal transformation products (Figure 2, Sections S8 and S9) that were significantly upregulated in fungal treatments compared to unexposed live fungal controls. Ten products matched those previously reported for HMMM in activated sludge; matches were determined using MS^2^ fragment information (see Section S8).^26^ The pathway described in the previous study involves hydrolysis of methoxy groups and *N*-methylol transformation to free amines or oxidation to aldehydes; the latter was only observed in the presence of activated sludge biomass.^26^ We similarly observed sequential oxidation of *N*-methylol to aldehydes in our fungal treatment samples, broadening evidence that this mechanism is biological.

We detected 10 novel metabolites (annotated MS^1^ and MS^2^ spectra with proposed fragmentation in Section S9). Eight metabolites exhibited at least one of the fragments previously noted as common to products with an intact triaminotriazine ring ([M + H]^+^*m*/*z* values: 151.0727, 163.0731, and 177.0889).^26^ We propose a novel product with a carboxylate group (TP169), albeit at a Level 3b confidence based on in-source fragmentation and chemical formula. Previous studies have not observed further oxidation from aldehydes to carboxylates,^21,26^ but extracellular oxidative enzymes or the intracellular enzyme superfamily cytochrome P450 could explain an additional level of oxidation. Of particular interest are two sets of isomers: one isomer of the previously reported^26^ TP363 and one entirely new set (TP335). These isomers have the same chemical formulas, similar (but not identical) fragmentation, and retention times within 1 min of each proposed isomer. Isomers are environmentally important because they can be selectively metabolized; have different toxicities; have different levels of bioaccumulation; and partition differently in soil, water, and air.^55−59^ Further study of HMMM isomer metabolites is needed to understand environmental fate and toxicity.

Notably, HMMM transformed to melamine in fungal treatment samples, indicating near-complete breakdown. Identification of melamine was confirmed to Level 1 confidence via a reference standard (Sections S8 and S9). Melamine could potentially be acetylated to form TP169, or TP169 could be decarboxylated to form melamine; aromatic amine N-acetylation and decarboxylation pathways in fungi have been reported previously.^60,61^ Nevertheless, melamine is poorly removed by conventional wastewater treatment, though bacteria able to metabolize melamine have been isolated.^62−65^ To our knowledge, there are no studies on fungal metabolism of melamine, though fungi can mineralize compounds with structurally similar triazine rings like hexahydro-1,3,5-trinitro-1,3,5-triazine (RDX).^66^ Future studies should explore the possibility of melamine ring cleavage, but given demonstrated recalcitrance in other organisms, melamine could be a “dead-end” product in biological removal. This has important toxicity implications, as melamine in pet foods and baby milk formula caused renal failure in animals and infants, respectively.^67,68^

All metabolites from biomass-extracted samples were present in extracellular samples, but there were extracellular metabolites that were not confirmed in biomass-extracted samples (i.e., insufficient upregulated features to confirm at the retention time). This result could indicate intracellular metabolism followed by release into media, or extracellular enzymes acting on HMMM/HMMM metabolites. Alternatively, released metabolites could be transformed via biologically mediated hydrolysis as suggested by Alhelou et al.^26^

### Acetanilide Products and an Underappreciated Amino Acid Conjugation Pathway

**Figure 3:**
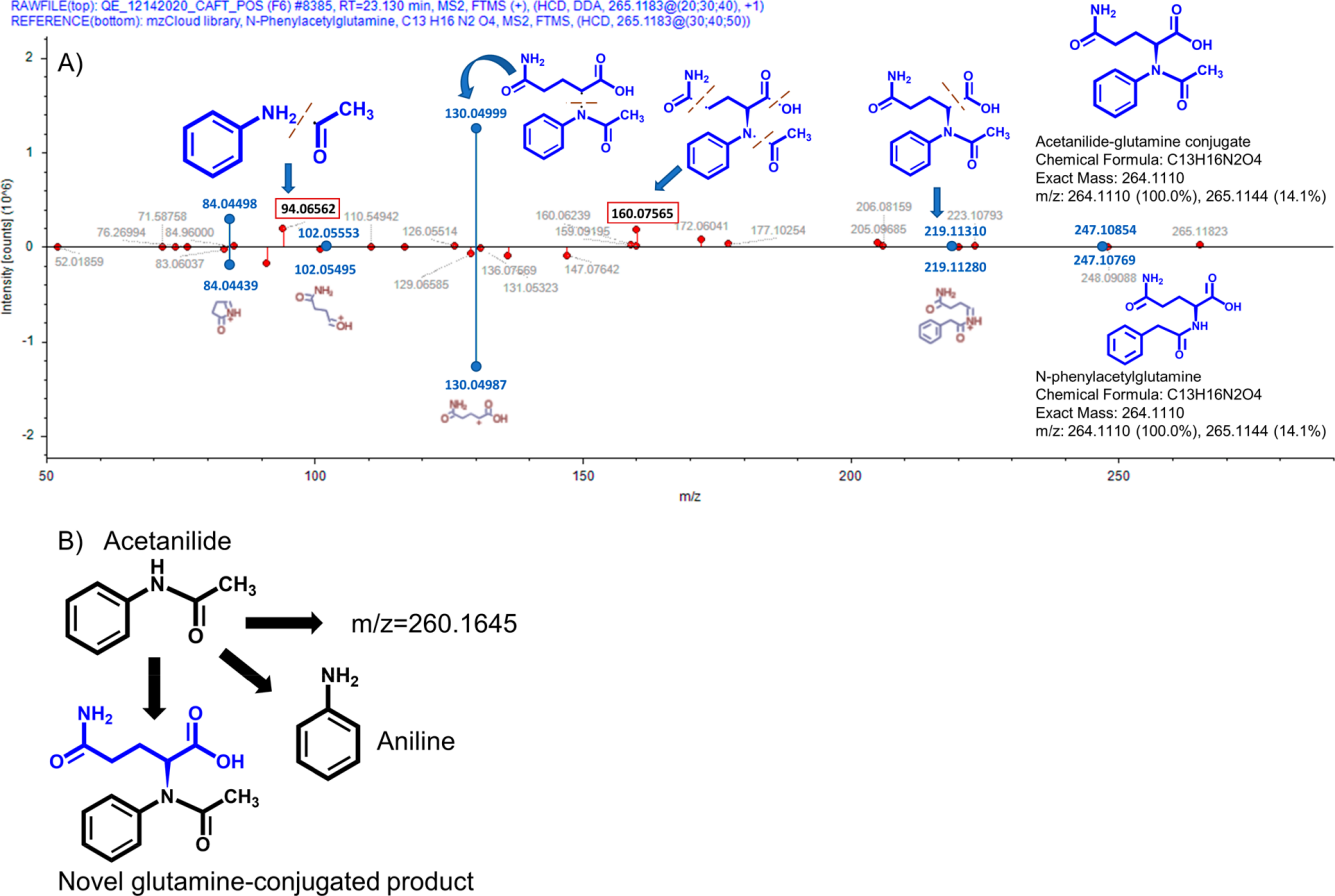
(A) Annotated MS^2^ spectra comparison for putative glutamine-conjugated product (top) and mzCloud *N*-phenylacetylglutamine spectra (bottom). Blue ● indicate matches in fragments, and red ● indicate discrepancies between spectra. The [M + H]^+^*m*/*z* = 94.0656 and *m*/*z* = 160.0757 fragments support the proposed position of the nitrogen in the glutamine-conjugated product. (B) Proposed acetanilide *T. versicolor* metabolism pathway. The [M + H]^+^*m*/*z* = 260.1645 product was significantly upregulated in fungal treatments, but no chemical structure could be proposed based on the data due to multiple chemical formula predictions within 5 ppm error. Though no other significantly upregulated transformation products were detected, it is possible that the glutamine-conjugated product and aniline are not “dead-end” products and could undergo further transformation or mineralization.

Compound Discoverer analysis of acetanilide revealed three upregulated products in the fungal treatments: aniline and two novel metabolites (one novel metabolite, [M + H]^+^*m*/*z* = 260.1645, had insufficient data to propose a structure). Aniline is a known minor human metabolite of acetanilide and is more toxic to humans than acetanilide.^69^ Aniline, along with chlorinated or substituted anilines, is also a known aquatic toxicant.^70−73^ However, there is evidence for aniline degradation by other fungi; it is possible that aniline is not a “dead-end” metabolite.^74,75^ Most notably, we propose a novel glutamine-conjugated product. This product has an MS^2^ fragmentation similar to that of its closest database match, *N*-phenylacetylglutamine (89%; mzCloud reference 541), but the [M + H]^+^*m*/*z* = 94.0656 fragment implicates preservation of the aniline group (Figure 3). The MS^2^ spectrum lacks some of the common features for *N*-phenylacetylglutamine ([M + H]^+^*m*/*z*: 91.054, 136.076, 147.076; HMDB^76^ reference: HMDB0006344). Additionally, we did not detect phenylacetate in either positive or negative ionization mode. If the product were *N*-phenylacetylglutamine, we would expect to detect phenylacetate, as *N*-phenylacetylglutamine forms via glutamine conjugation with phenylacetate.^77^ The collective evidence implicates a novel glutamine-conjugated product.

To our knowledge, this is the first detection of a glutamine-conjugated product in *T. versicolor* and white rot fungi more broadly. The literature on amino acid conjugation as a metabolic mechanism in fungi is remarkably sparse; we found only three studies discussing amino acid-conjugated products identified in fungi. Two studies investigated natural products discovery, with one study on amino acid-conjugated anthraquinones produced by a marine-derived *Penicillium* fungus^78^ and one on natural colorant compounds produced by *Penicillium marneffei*.^79^ The third study implicated glutamic acid conjugation with *p-*substituted benzoic acid by an endophytic fungus, *Xylaria arbuscula*, but had little discussion on the significance of the transformation product.^80^ There are numerous potential reasons for this paucity in the literature. Many studies have investigated the diverse metabolism of fungi, but attention is mainly on carbohydrate-active enzymes^81^ (i.e., CAZy; enzymes that synthesize/metabolize carbohydrates such as those acting on plant cellulose, lignin, and hemicellulose) critical in carbon cycling and contaminant removal (e.g., ligninolytic peroxidases).^82,83^ Additionally, molecular techniques (e.g., sequencing) have only been applied to fungi somewhat recently.^84−86^ As molecular technology advances, studies can investigate the intricacies of fungal metabolism.

Although amino acid conjugation is yet underappreciated in fungi, a growing number of studies recognize the mechanism’s importance in plants. Amino acid conjugates such as jasmonoyl isoleucine can have key roles in stress response, plant defense, and plant growth.^87^ Plants also form amino acid conjugates with xenobiotics, as evidenced by amino acid conjugation with the anticorrosive benzotriazole, the tire rubber vulcanizer 2-mercaptobenzothiazole, and the plasticizer di-*n*-butyl phthalate in *Arabidopsis*.^88−90^ Some of these conjugates are structurally analogous to natural plant compounds such as indoles, which have critical functions in hormone storage. More work is necessary to determine the function of the amino acid-conjugated product discovered in this study.

### Environmental Implications

Biotransformation and metabolite identification studies are urgently needed to fully understand fate and treatment options of TWCs. White rot fungi could be present *in situ* or bioaugmented into green infrastructure (e.g., bioretention) to facilitate removal of recalcitrant organic contaminants like TWCs and restore sorption capacity of media. This is the first study to report fungal removal of the environmentally relevant TWCs acetanilide and HMMM and the first detection of a glutamine-conjugated product from fungal biomass. Full biodegradation of some TWCs may be possible. However, toxic byproducts may form as a result—as demonstrated by the formation of melamine from HMMM. 1,3-Diphenylguanidine was not removed and should be further studied for toxicity and removal by other organisms. Additionally, stormwater contains a variety of contaminants, including heavy metals from TRWPs, that were not examined in this study; heavy metals can induce white rot fungi oxidative enzymes or be toxic in excess,^91−94^ warranting future study. There is mounting evidence that amino acid conjugates can impact key biological functions or have utility as natural products. Many organisms can form amino acid-conjugated products with xenobiotics, which has environmental fate and toxicity implications. For example, amino acid conjugates may undergo deconjugation reactions, which could re-release the parent into the environment or within the body if consumed by humans.^95,96^ Future studies should investigate fungal amino acid conjugation from different angles: fundamental fungal metabolism, xenobiotic metabolism pathways, natural products discovery, etc. Nonetheless, this study is a first step in understanding the potential role of fungi in degrading toxic/recalcitrant organic contaminants found in stormwater and opens future research opportunities on fungal amino acid conjugation.

## Supporting Information Available

Additional experimental and analytical method details, sorption to biomass figure, data for 1,3-diphenylguanidine, kinetics and absolute concentration information, high-resolution mass spectrometry data, and annotated MS^1^ and MS^2^ spectra (PDF)

## Notes

*Data Availability Statement.* The underlying data for this work are available in two online repositories. Raw kinetics data and associated metadata can be found in the Iowa Research Online (IRO) repository under an Open Data Commons Attribution License (ODC-By).^97^ HRMS and Compound Discoverer data are available in the Mass Spectrometry Interactive Virtual Environment (MassIVE) database under a CC0 1.0 Universal (CC0 1.0) license (MassIVE ID: MSV000088889).^98^ These underlying data are available free of charge and do not require registration to download.
