# Supplementary material for: White Rot Fungi Produce Novel Tire Wear Compound Metabolites and Reveal Underappreciated Amino Acid Conjugation Pathways
Source: Environ Sci Technol Lett. 2022 Mar 18;9(5):391–9. doi: 10.1021/acs.estlett.2c00114 (PMC9100321; doi:10.1021/acs.estlett.2c00114)
Supplement: Supplementary file 1 — ez2c00114_si_001.pdf [file ez2c00114_si_001.pdf]

Supporting Information

White Rot Fungi Produce Novel Tire Wear Compound Metabolites and Reveal Underappreciated Amino Acid Conjugation Pathways

Erica A. Wiener[§,¶] *and Gregory H. LeFevre*[§,¶,*]

[§]Department of Civil & Environmental Engineering, University of Iowa, 4105 Seamans Center, Iowa City, IA 52242, United States; [¶]IIHR-Hydroscience &Engineering, 100 C. Maxwell Stanley Hydraulics Laboratory, Iowa City, IA 52242, United States

***Corresponding Author:**
GHL: gregory-lefevre@uiowa.edu; Phone: 319-335-5655; Fax: 319-335-5660; 4105 Seamans Center for Engineering, University of Iowa, Iowa City IA, United States

Includes: 48 total pages, 31 Figures, and 8 Tables.

Table of Contents

SECTION S1: CHEMICALS S3
SECTION S2 FUNGAL CULTURE AND MAINTENANCE PROCEDURES S4
SECTION S3 BATCH EXPERIMENT DETAILED METHODOLOGY AND QUALITY CONTROL . S5
SECTION S4 METHODOLOGY FOR METABOLITE EXTRACTION FROM BIOMASS S6
SECTION S5 CHROMATOGRAPHY METHODS S7
SECTION S6 METHODOLOGY FOR METABOLITE ANALYSIS USING COMPOUND DISCOVERER S9
FIGURE S1 SORPTION TO BIOMASS EXPERIMENT S12
FIGURE S2 BATCH EXPERIMENT FOR 1,3-DIPHENYLGUANIDINE S12
SECTION S7 KINETICS DATA AND CALCULATIONS S13
SECTION S8 DATA FROM ORBITRAP AND COMPOUND DISCOVERER S14
SECTION S9 ANNOTATED SPECTRA FOR HMMM S19

SECTION S1: CHEMICALS

Target analytes/standards were dissolved in reagent grade methanol to make individual concentrated stock solutions. Stock solutions were stored in amber glass vials at -20 °C until use. Solvents used for chromatography (water, methanol, formic acid) were Optima LC/MS grade and purchased from Fisher Scientific.

Table S1. List of chemicals used in this work, grouped by use.

Chemical Name	CAS Number	Purity	Manufacturer	Use
Calcium chloride	10043-52-4	96%	Acros Organics	Synthetic stormwater
Magnesium chloride hexahydrate	7791-18-6	ACS	Fisher Scientific	
Sodium sulfate	7757-82-6	ACS	Sigma Aldrich	
Sodium nitrate	7631-99-4	ACS	Fisher	
Ammonium chloride	12125-02-9	ACS	Research Products International	
Sodium bicarbonate	144-55-8	ACS	EMD	
Sodium phosphate dibasic	7558-79-4	ACS	Research Products International	
Acetanilide	103-84-4	99+%	Acros Organics	Target analyte or standard
1,3-diphenylguanidine	102-06-7	99+%	Alfa Aesar	
Hexamethoxymethylmelamine	3089-11-0	98%	TCI America	
Melamine	108-78-1	99%	Acros Organics	
Malt extract broth	N/A	N/A	Research Products International	Fungal Growth Medium

Sodium azide[1]	26628-22-8	ACS	Fisher Scientific	Sorption control

[1]NOTE: Sodium azide (NaN_3) is acutely hazardous, and all recommended safety procedure should be followed, especially when working with the pure, powdered form.

SECTION S2 FUNGAL CULTURE AND MAINTENANCE PROCEDURES

Fungal maintenance. Dr. Jordyn Wolfand (University of Portland) graciously provided cultures of *Trametes versicolor* (ATCC#42530). To maintain the fungi, 100 μL of homogenized *T. versicolor* in 2% liquid malt extract media was plated onto malt extract agar. Both malt extract media and malt extract agar were made according to manufacturer instructions. Plates were allowed to incubate at room temperature for 1-3 weeks (or until biomass was well-established) and stored at 4 °C. Fresh plate cultures were made monthly. All culture work was conducted in a UV-sterilized and ethanol-wiped laminar flow biosafety cabinet. After any work with fungal cultures, the biosafety cabinet was decontaminated using 10% (v/v) household bleach for 30 minutes, then wiped with ethanol and RO water. Reusable loops were also decontaminated using dilute bleach solution. A US Department of Agriculture Permit to Move Live Plant Pests, Noxious Weeds, and Soil was obtained for this work.

Pre-experiment cultures. Using a sterilized loop, fungal biomass was transferred from plate cultures into 30 mL of liquid malt extract media in sterile falcon tubes. Cultures were incubated for five to seven days in the dark on a platform shaker set to 80 rpm. Cultures were checked for contamination (cloudy media or non-white fungal biomass). To homogenize the liquid cultures, an Omni Tissue Master 125 was used to blend the cultures for 3-5 minutes or until visibly homogeneous.

SECTION S3 BATCH EXPERIMENT DETAILED METHODOLOGY AND QUALITY CONTROL

Experimental Setup. To ensure homogeneity across replicates, a "master mix" was created by nominally spiking 30 µM target analyte (HMMM or acetanilide) into pH 7 synthetic stormwater. Methanol introduced from spiking the target analyte into the master mix was less than 0.05% of the final volume. Abiotic controls contained 20 mL of "master mix", and fungal treatments contained 19.2 mL master mix and 0.8 mL homogenized *T. versicolor* inoculum. Sample containers were 125 mL clear serum bottles capped with PTFE-coated butyl rubber septa and aluminum crimp caps, all sterilized by autoclave. To minimize light exposure, sample bottles were placed in a cardboard box and covered in foil on a platform shaker at 80 rpm for the duration of the experiment.

Sampling. 3 mL Luer-Lok disposable syringes with sterile needles were used to remove 1.2 mL of sample and filtered into amber glass autosampler vials using 0.2 µm PTFE filters. All sampling was conducted in a UV-sterilized and ethanol-wiped laminar flow biosafety cabinet. All samples were stored in the freezer at -20 °C prior to analysis.

Sorption to Biomass Experiment. To test sorption of HMMM to fungal biomass, a paired batch experiment was conducted with fungal treatments (*T. versicolor* in synthetic stormwater with 30 µM HMMM) and a fungal sorption control. Fungal sorption controls contained 19.1 mL of synthetic stormwater and 0.8 mL of fungal inoculum. At days 0, 3, 7, and 15, fungal sorption was tested by adding 40 µL of 5M sodium azide to duplicate sacrificial sorption controls. Upon adding the sodium azide, the bottles were mixed for one hour at 80 rpm on the platform shaker. At one hour of mixing, HMMM was spiked into the sorption control bottles to attain 30 µM HMMM. All bottles including the fungal treatment were sampled after another hour of mixing according to the sampling procedure described previously. To confirm that

sodium azide does not react with HMMM, abiotic azide controls containing 19.96 mL HMMM master mix and 40 µL of 5M sodium azide were sampled (non-sacrificial) at days 0 and 3.

SECTION S4 METHODOLOGY FOR METABOLITE EXTRACTION FROM BIOMASS

Exposed and unexposed fungi were compared to determine acetanilide and HMMM metabolites. Exposed fungi samples contained 19.2mL of nominal 30 µM target analyte in synthetic stormwater and 0.8 ml homogenized *T. versicolor*. Unexposed fungi samples contained 19.2 mL synthetic stormwater and 0.8 mL homogenized *T. versicolor*. To sample, fungal biomass was removed using a flame-sterilized metal loop and transferred to a 2 mL microcentrifuge tube and frozen overnight at -80 °C. Frozen samples were lyophilized (Labconco Freezone 6) to preserve integrity of biomass prior to metabolite extraction.

The adapted metabolite extraction method is as follows: 1 mL of 1:1 (v/v) methanol/water solution and a single sterile stainless steel homogenization bead (5 mm) were added to the lyophilized samples. To begin lysing biomass, samples were frozen for 30 minutes at -80 °C and thawed before placing on a Retsch mixer mill bead homogenizer (MM 400) for 5 minutes at 30 Hz. Metabolites were extracted into solution through subsequent mechanical agitation; samples were sonicated for 10 minutes, vortexed for 1 minute, and centrifuged for 10 minutes at 10000 × g. After extraction, the supernatant was filtered with a 0.2 µm PTFE filter into an amber autosampler vial and stored at -20 °C until analysis.

SECTION S5 CHROMATOGRAPHY METHODS

HPLC-DAD analysis. To develop analytical methods for HPLC-DAD, the peak wavelength of the analyte was determined using a spectrophotometer. Isocratic methods were developed with the parameters in Table S2. The column used for HPLC-DAD analysis was a Higgins Analytical Sprite Targa C18 (40 x 2.1 mm, 5 µm) with a guard column of the same material.

Table S2. HPLC-DAD isocratic methods for acetanilide and HMMM kinetics

Parameter	Parameter value for acetanilide	Parameter value for HMMM
Peak wavelength	238.5 nm	215 nm
Column temperature	50 ℃	50 ℃
Injection volume	10 µL	20 µL
Pump Flow	0.6 mL/min	0.5 mL/min
Method Length	10 minutes	10 minutes
Mobile Phases	85% water with 0.1% formic acid, 15% methanol with 0.1% formic acid	50% water with 0.1% formic acid, 50% methanol with 0.1% formic acid

High resolution spectrometry analysis. Individual replicate samples were run with polarity-switching MS (both positive and negative ionization in a single method run). For data-driven MS^2 ($ddMS^2$) analysis, composite samples of the replicates were created using equal volume aliquots from the individual replicate samples to account for biological variability. The composite samples were run for $ddMS^2$ only (one run in positive and one run in negative mode.) Parameters for polarity-switching and $ddMS^2$ runs are described in Table S3. Both biomass-extracted samples and extracellular samples were run at the HRMS for HMMM; only biomass-extracted samples were run for acetanilide. The chromatography column used for metabolite identification was an Agilent Eclipse Plus C18 (4.6 x 150 mm, 5 µm) with a guard column of the same material.

Table S3. Settings for the Q Exactive Orbitrap MS runs. An inclusion list based on features of interest from Compound Discoverer was used for one of the runs with extracellular samples and melamine standard confirmation. An exclusion list for all ddMS^2 runs was generated by HRMS facility staff.

Parameter	Parameter value (polarity switching)	Parameter value (ddMS^2)
Column temperature	25 °C	25 °C
Injection volume	10 μL	10 μL
Pump Flow	0.6 mL/min	0.6 mL/min
Method Length	50 minutes	50 minutes
Mobile Phases	Water with 0.1% Formic Acid, Methanol with 0.1% Formic Acid	Water with 0.1% Formic Acid, Methanol with 0.1% Formic Acid
Resolution	70000	70000
Scan range	70 to 1000 m/z	70 to 1000 m/z
dd-MS^2 microscans	N/A	1 at resolution 17,500 with 5 loop counts. “Top N” or the maximum number of abundant ions to trigger a scan is 5. 200-2000 m/z for the scan range and a 0.4 m/z isolation window
Normalized collision energy	N/A	20, 30, 40
Dynamic exclusion (temporarily puts a mass into an exclusion list)	None	5.0 s
Spectrum data type	Profile	Profile

Table S4. Gradient method for HRMS runs on the Q Exactive. The flow rate was 0.6 mL/min throughout, with the first minute sent to waste.

Time (min)	A (%)	B (%)
0	95	5
1	95	5
26	50	50
39	5	95
44	5	95
45	95	5
50	95	5

SECTION S6 METHODOLOGY FOR METABOLITE ANALYSIS USING COMPOUND DISCOVERER

In Compound Discover, the workflow "Untargeted Metabolomics with Statistics Detect Unknowns with ID using Online Databases and mzLogic" was used to identify features that were significantly up- or down-regulated between the exposed fungal treatment and unexposed live fungal control. The Log2 Fold Change is calculated by Compound Discoverer using a one-way ANOVA with a Tukey post-hoc test and P-values adjusted by a Benjamini-Hochberg algorithm. To make metabolite analysis more robust, adjustments were made to the Compound Discoverer workflow. The following nodes were added in the Workflows tab: Calculate mass defect, map to KEGG pathways, mzVault, and assign compound annotation. The predict composition node was modified to remove Br, Cl, and P, and the fragment tolerance was increased from 5 ppm to 10 ppm. ChemSpider was added to the search databases.

The following filters were used for post-processing of Compound Discoverer data: "background is false", "log2 fold change greater than or equal to 2 OR log2 fold change less than or equal to -2", and "p-value less than or equal to 0.05". The filtered results were sorted by retention time and features that were within 0.5 minutes of the proposed compound were grouped as likely in-source fragments of that compound. Xcalibur Freestyle was used for mass spectral analysis of putative metabolites. Likely fragment compounds and adducts from Compound Discoverer were confirmed in the mass spectra from the raw files.

The Schymanski framework was used to communicate confidence levels for metabolite identification. For this study, we are specifying our usage of Levels 2a and 2b and defining sub-levels within Level 3 confidence. We note that defining sub-levels for Level 3 is only for providing additional clarity to our results; Schymanski et al recommend defining sublevels on a per-study basis as necessary. Therefore, we do not necessarily recommend these definitions for

Level 3a and 3b to be used outside of this context. Related future studies should carefully consider how confidence levels apply to study-specific results.

Table S5. Description of our use of the Schymanski framework, including study-specific sublevels.

Confidence Level	Description from Schymanski et al	Justification in this use case
Level 1	Confirmed structure by reference standard	N/A; same as in the original framework
Level 2a	Probable structure by library spectrum match	For HMMM, any products that matched in-source and/or MS^2 fragments with Alhelou et al or Johannes et al.<br><br>We did not identify any acetanilide structures to a Level 2a.
Level 2b	Probable structure by diagnostic evidence	For HMMM, any structures that had a partial match to Alhelou et al or Johannes et al. (e.g., 1/5 of the most abundant MS^2 features from our data match) but did not have certain fragments listed in the study or had additional fragments not listed in the study.<br><br>For acetanilide, the Level 2b classification was used for the putative glutamine conjugate due to closest match with a similar structure in reference libraries, with MS^2 evidence to support the proposed structure.
Level 3a	Tentative candidate(s)	The following situations were identified as 3a for HMMM products:<br>• Structural isomers of previously discovered or novel products were classified as Level 3a when supported by MS^2 data and within 1 minute in retention time difference<br>• Evidence of a sodium adduct as the base peak (based on Alhelou et al or evidence from MS^1/MS^2). In these cases, the transformation product may not appear in Compound Discoverer since the

		adduct dominated in intensity at that retention time.<br>• Product is previously identified but no MS^2 fragments in our data match with the literature<br>• For novel products, structures that could be resolved using MS^2 fragmentation based on ability to resolve three of the five most abundant fragments or 60% of fragments with relative abundance of 10% or greater, whichever was larger
Level 3b	Tentative candidate(s)	Products were classified as a Level 3b when a structure could be proposed based on the chemical formula, but there was insufficient evidence from the MS^2 data to increase confidence in the identification. Examples include:<br>• MS^2 available for likely in-source fragments but not the base peak<br>• MS^1 has informative in-source fragments that allow for structure proposal but no MS^2 is available
Level 4	Unequivocal molecular formula	N/A; same as in the original framework
Level 5	Exact mass of interest	Compounds that had a log2 Fold change magnitude greater than 2, but fell under one of the following cases:<br>• The base peak could not be identified from the data (e.g., only one feature showed up in Compound Discoverer and the MS data were ambiguous)<br>• No unequivocal formula could be calculated with error < 5 ppm

FIGURE S1 SORPTION TO BIOMASS EXPERIMENT

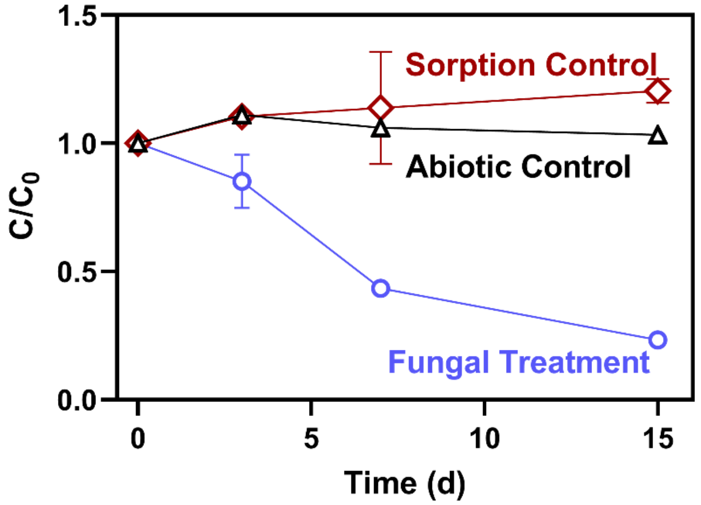

Figure S1. Concentration of HMMM over time divided by initial concentration for sorption to fungal biomass (red diamonds), fungal treatment (blue circles), and abiotic controls (black triangles). Error bars represent standard error of duplicate samples about the mean, with some error bars obscured by plot markers. There was no significant loss of HMMM in either the abiotic controls or sorption controls ($p=0.9559$ and $p=0.1969$, respectively).

FIGURE S2 BATCH EXPERIMENT FOR 1,3-DIPHENYLGUANIDINE

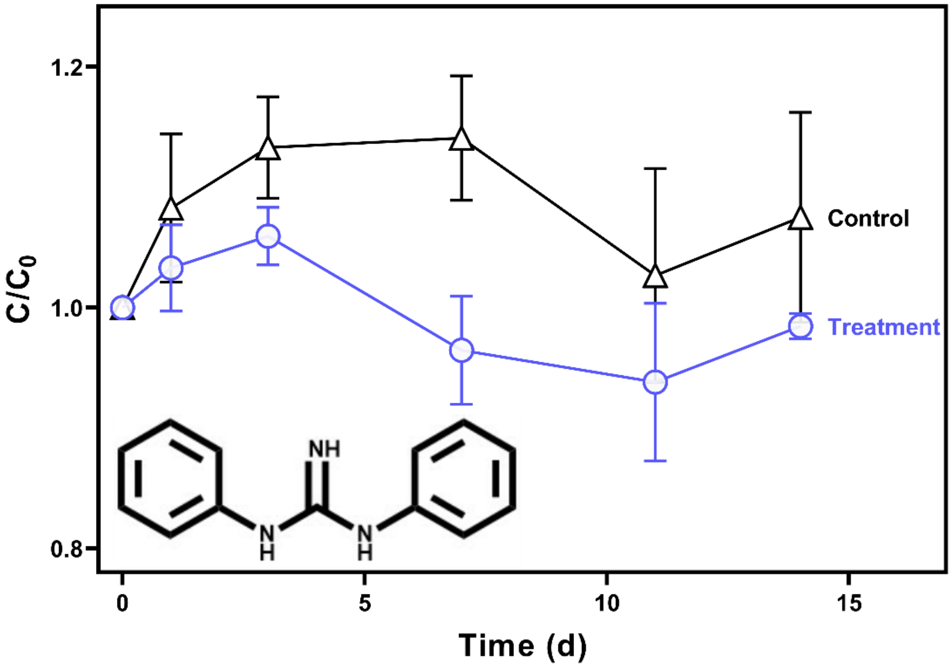

Figure S2. Concentration of 1,3-diphenylguanidine at time t divided by initial concentration for abiotic controls (black triangles) and fungal treatments (blue circles). Error bars represent standard error about the mean for duplicate samples. Some error bars are small and obscured by

the data symbols. There was no significant loss in either the treatments or controls (p=0.0999 and 0.9389, respectively).

SECTION S7 KINETICS DATA AND CALCULATIONS

Table S6. Calculated R^2 values for different rate order kinetics models for acetanilide and HMMM. In both cases, a first-order model best describes the data.

Compound	Rate Order	R^2
Acetanilide	Zero	0.9181
	**First**	**0.9531**
	Second	0.9237
Hexamethoxymethylmelamine	Zero	0.8521
	**First**	**0.9165**
	Second	0.8719

Table S7. Absolute concentrations of acetanilide and HMMM for kinetics experiments, calculated using a 5-point standard curve (acetanilide R^2=0.998; HMMM R^2=0.988).

Compound	Time Point	Average Concentration in Controls (µM)	Standard Error (n=3)	Average Concentration in Treatments (µM)	Standard Error (n=3)
Acetanilide	0	24.5	0.223	21.7	1.187
	3	25.2	0.404	17.9	0.657
	7	26.8	0.6	8.2	1.602
	15	25.1	0.2	4.4	0.189
HMMM	0	12.0	0.213	12.6	0.228
	3	12.1	0.180	10.5	0.855
	8	11.3	0.237	5.90	0.314
	15	11.4	0.069	3.65	0.065

SECTION S8 DATA FROM ORBITRAP AND COMPOUND DISCOVERER

Table S8. HRMS data for biomass-extracted fungal treatment samples with acetanilide. Accurate masses, proposed formulas, and fragment ions listed as $[M+H]^+$ are recorded from raw data. Log2 Fold Change values are recorded from Compound Discoverer (treatment over controls).

Metabolite	Proposed Structure	Proposed Formula	Retention Time (min)	Accurate mass (m/z)	Delta (ppm)	Confidence Level	Log2 Fold Change	Fragments		
								Fragment Ions (m/z)	Delta (ppm)	Molecular formula
Acetanilide	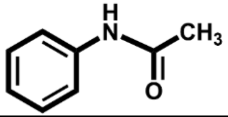	C8H10NO	21.96	136.0757	-0.05	Parent		94.0656	4.77	C6H8N
Product 260	N/A	C16H22O2N<br><br>Other predictions:<br>C9H22N7S<br>C14H20ON4	36.79	260.1643	-0.65	Level 5	6.27	242.1536	-1.37	C16H20ON
								224.1430	-1.79	C16H18N
								214.1591	0.43	C15H20N
								158.0959	-3.32	C11H12N
								144.0807	-0.71	C10H10N
								132.0808	0.49	C9H10N
								130.0655	2.81	C9H8N
								118.0654	1.99	C8H8N
								106.0655	3.22	C7H8N
								93.0711		
Acetanilide glutamine-conjugate	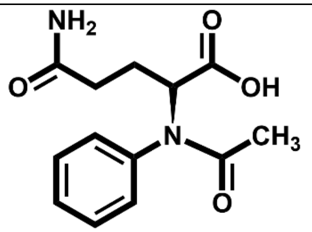	C13H17O4N2	23.12	265.1182	-0.22	Level 2b	9.46	205.0968	-1.5	C11H13O2N2
								177.1025	1.69	C10H13ON2
								172.0604	4.52	C12H14N
								160.0757	-0.23	C10H10ON
								130.05	0.91	C5H8O3N
								94.0656	5.26	C6H8N
								84.045		
Aniline	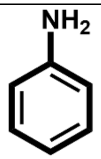	C6H8N	5.43	94.0656	5.01	Level 2a	9.23			

Table S9. HRMS data for fungal treatment samples with HMMM, with accurate masses, proposed formulas, and fragment ions listed as $[M+H]^+$ as recorded from raw data. Retention times are listed for extracellular samples unless marked with an asterisk. Asterisk marks indicate data from biomass-extracted samples. Log2 Fold Change values are recorded from Compound Discoverer (treatment over controls). Matched fragments from the literature are displayed where applicable in blue text for non-adduct fragments from Alhelou et al or Johannessen et al (marked with ⁑).

Metabolite	Proposed Structure	Proposed Formula	Retention Time (min)	Accurate mass (m/z)	Delta (ppm)	Confidence Level	Log2 Fold Change	Fragments			
								Matching Literature Fragment ions (m/z)	Fragment ions (m/z)	Delta (ppm)	Molecular formula
Hexamethoxy methyl-melamine	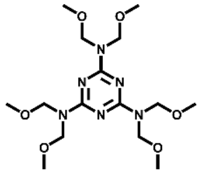	C15H31N6O6	33.45	391.2289	-2.75	Parent compound		359.2056	359.2032	0.57	C16H29O6N3
								315.1788	315.1772	-0.90	C12H23O4N6
								283.1520	283.1509	-1.00	C11H19O3N6
								239.1263	239.1248	-1.18	C9H15O2N6
								207.1003	207.0987	-0.34	C8H11ON6
								177.0889	177.0882	-0.19	C7H9N6
TP377	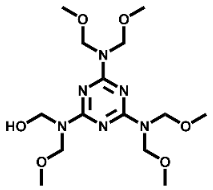	C14H29N6O6	30.71	377.2139	-1.01	Level 2a (8/13 matching non-adduct fragments)	10.04	345.1890	345.1869	-3.49	C13H25O5N6
								301.1628	301.1617	-0.66	C11H21O4N6
								283.1531	283.1512	-0.57	C11H19O3N6
								269.1369	269.1355	-0.62	C10H17O3N6
								239.1262	239.1250	-0.42	C9H15O2N6
								207.1000	207.0989	0.03	C8H11ON6
								177.0885	177.0883	0.16	C7H9N6
								163.0736	163.0727	0.04	C6H7N6
TP363 isomer	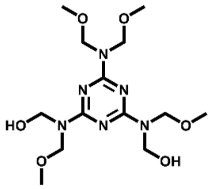	C13H27O6N6	27.49	363.1978	-2.41	Level 3a (4/8 matching non-adduct fragments)	9.85		269.1355	-0.62	C10H17O3N6
								239.1258	239.1251	0.16	C9H15O2N6
									225.1090	-2.16	C8H13O2N6
								207.0993	207.0984	-2.40	C8H11ON6
									193.0834	0.72	C7H9ON6
								177.0889	177.0882	-0.96	C7H9N6
								163.0732	163.0723	-2.30	C6H7N6
TP363		C13H27O6N6	26.87	363.1984	-0.72	Level 3a (6/8 matching non-adduct fragments)	10.21		331.1722	-0.99	C12H23O5N6
								283.1521	283.1512	-0.38	C11H19O3N6
								255.1208	255.1200	-0.24	C9H15O3N6

	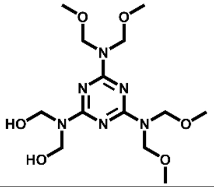							239.1258	239.1251	-0.15	C9H15O2N6
								207.0993	207.0989	0.22	C8H11ON6
								177.0889	177.0883	0.1	C7H9N6
								163.0732	163.0727	0.15	C6H7N6
Novel TP333	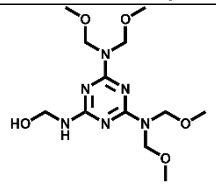	C12H25O5N6	26.26	333.1874	-1.94	Level 3a	7.54		239.1247	-1.70	C9H15O2N6
									225.1087	-3.18	C8H13O2N6
									209.1147	0.93	C8H13ON6
									195.0987	-0.98	C7H11ON6
									177.0879	-2.34	C7H9N6
									163.0724	-1.64	C6H7N6
TetraMMM TP303	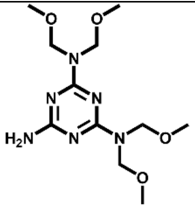	C11H23O4N6	24.11	303.1768	-0.74	Level 3a	9.08 (fold change for sodium adduct 324.15178)				
TP349	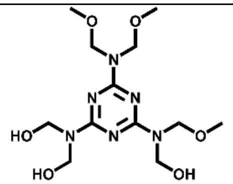	C12H25O6N6	23.17	349.1823	-0.76	Level 2b (2/3⁑ matching non-adduct fragments)	7.73		255.1194	-2.23	C9H15O3N6
									225.1088	-2.77	C8H13O2N6
									207.0986	-1.15	C8H11ON6
								⁑ 177.0882	177.0884	0.59	C7H9N6
									193.0820	-6.32	C7H9ON6
								⁑ 163.0731	163.0724	-1.92	C6H7N6
Novel TP335	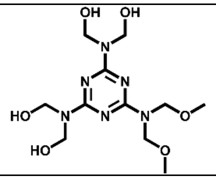	C11H23O6N6	19.97*	335.1674*	-0.01*	Level 3a	6.23*		225.1095	0.41	C8H13O2N6
									211.0935	-1.54	C7H11O2N6
									207.0989	0.03	C8H11ON6
									163.0728	0.88	C6H7N6
Novel TP335 isomer		C11H23O6N6	19.18*	335.1674*	0.09*	Level 3a	7.22* (fold change for sodium adduct 356.14175)		255.1194	-2.35	C9H15O3N6
									225.1101	3.06	C8H13O2N6
									211.0938	-0.02	C7H11O2N6
									193.0835	1.27	C7H9ON6
									177.0886	1.71	C7H9N6

	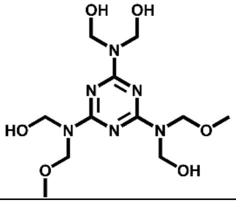								163.0727	0.32	C6H7N6
Novel TP289	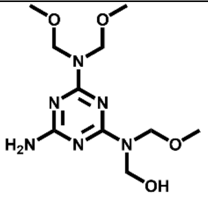	C10H21O4N6	19.15	289.1613	-1.95	Level 3a	8.35 (fold change for sodium adduct 311.1432)		195.0983	-2.78	C7H11ON6
									165.0881	-1.50	C6H9N6
									163.0727	0.23	C6H7N6
									151.0725	-1.27	C5H7N6
TriMMM TP259	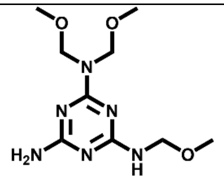	C9H19O3N6	15.46	259.1509	-1.70	Level 2a (4/4 matching non-adduct fragments)	7.2	227.1262	227.1242	-3.87	C8H15O2N6
								195.0997	195.0985	-2.08	C7H11ON6
								165.0891	165.0880	-1.96	C6H9N6
								151.0730	151.0726	-0.66	C5H7N6
TP243	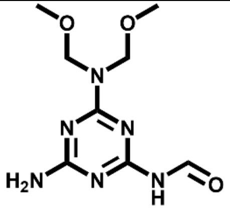	C8H15O3N6	14.37	243.1196	-1.63	Level 3a (0/5 matching non-adduct fragments)	6.48		195.0992	1.44	C7H11ON6
									165.0880	-2.05	C6H9N6
									151.0724	-1.47	C5H7N6
TP245	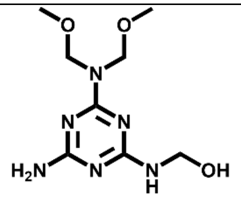	C8H17O3N6	11.96	245.1353	-1.49	Level 3a (2/2 matching non-adduct fragments)	6.23 (fold change for sodium adduct 267.1171)		227.1247	-1.85	C8H15O2N6
									195.0986	-1.69	C7H11ON6
								165.0883	165.0881	-1.4	C6H9N6
								151.0726	151.0725	-1.37	C5H7N6
Novel TP213	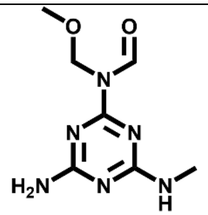	C7H13O2N6	11.42	213.1094	-0.14	Level 3b	4.00		165.0882	-0.85	C6H9N6
									151.0726	-0.36	C5H7N6

DiMMM TP215	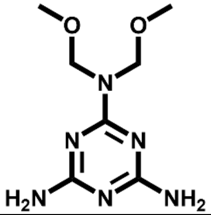	C7H15N6O2	10.35	215.1250	-0.55	Level 3a	6.02 (fold change for sodium adduct 237.1067)				
Novel TP231	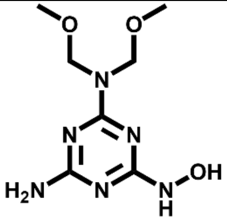	C7H15O3N6	8.90	231.1199	-0.46	Level 3a	4.58		213.1099<br>165.0882<br>151.0726	2.15<br>-0.57<br>-0.56	C7H13O2N6<br>C6H9N6<br>C5H7N6
Novel TP201	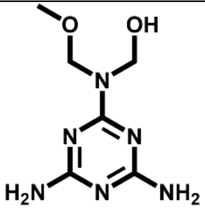	C6H13O2N6	7.2	201.1094	-0.40	Level 3b	6.46		139.0726	-0.61	C4H7N6
Novel TP169	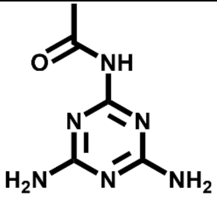	C5H9ON6	5.4	169.0831	-0.51	Level 3b	6.07				
Novel TP187	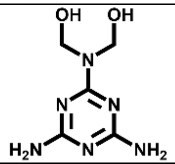	C5H11O2N6	4.46	187.0938	-0.17	Level 3a	5.52		139.0725	-0.94	C4H7N6
TP157	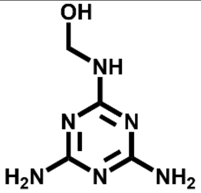	C4H9ON6	3.56	157.0832	0.05	Level 2a (1/2 matching fragments)	8.03	139.0729	139.0726<br>110.0463<br>97.0513	-0.61<br>1.92<br>4.09	C4H7N6<br>C3H4N5<br>C3H5N4

Melamine	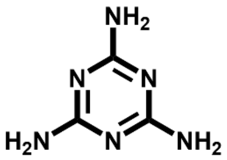	C3H7N6	2.94	127.0728	0.65	Level 1	8.29		85.0514	6.64	C2H5N4

SECTION S9 ANNOTATED SPECTRA FOR HMMM

This section includes annotated spectra (MS^1 and MS^2) for each proposed feature or transformation product for HMMM of Level 3 confidence and above. MS^1 spectra are displayed as average spectra for that retention time with a background subtraction of 0.2 minutes on either side of the peak. Anything labeled as an “in-source fragment” is based on the Compound Discoverer data within 0.5 minutes of that retention time. Note there is a retention time difference between biomass-extracted samples and extracellular samples; this is because the two sample sets were not analyzed in the same run and a different column (same brand and type) was used due to column failure.

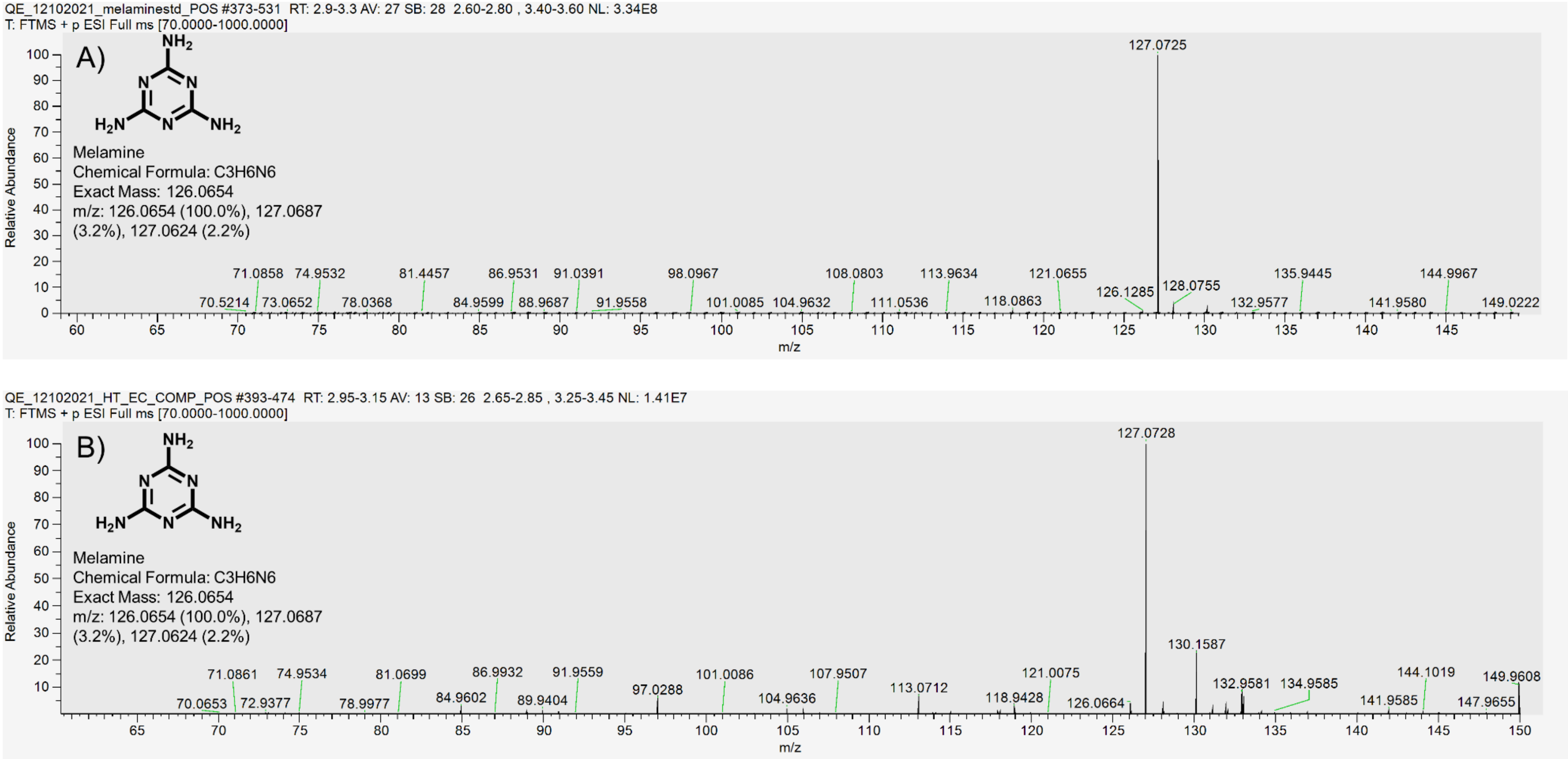

Figure S3. MS[1] spectra for the melamine reference standard (A) and the proposed transformation product melamine in fungal treatment extracellular samples (B). The differences in retention time between standard and treatment samples was 0.03 minutes.

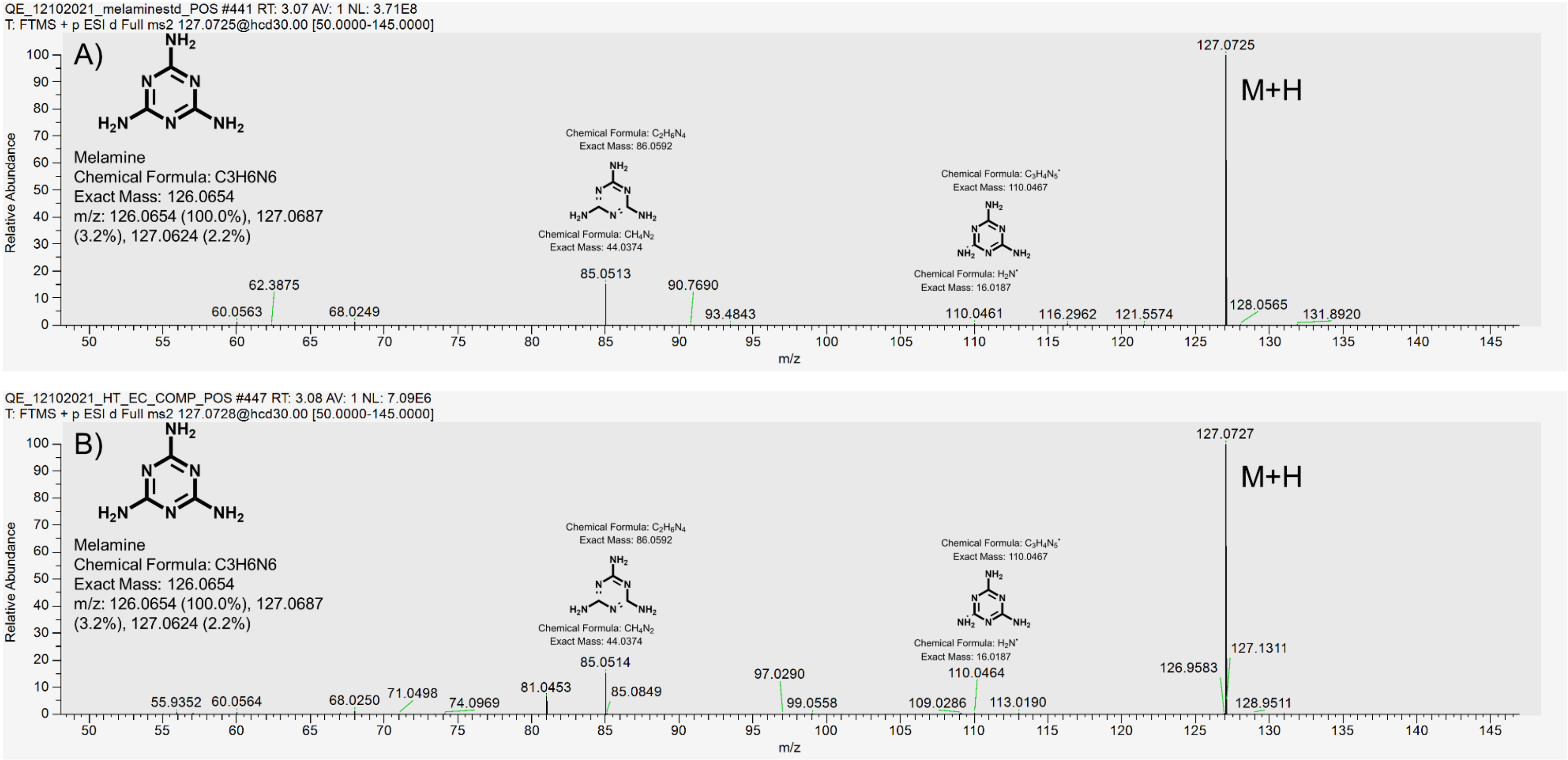

Figure S4. Annotated MS^2 spectra for melamine standard (A) and proposed transformation product melamine in fungal treatment extracellular samples (B). Fragmentation patterns are almost identical in the standard and treatment samples.

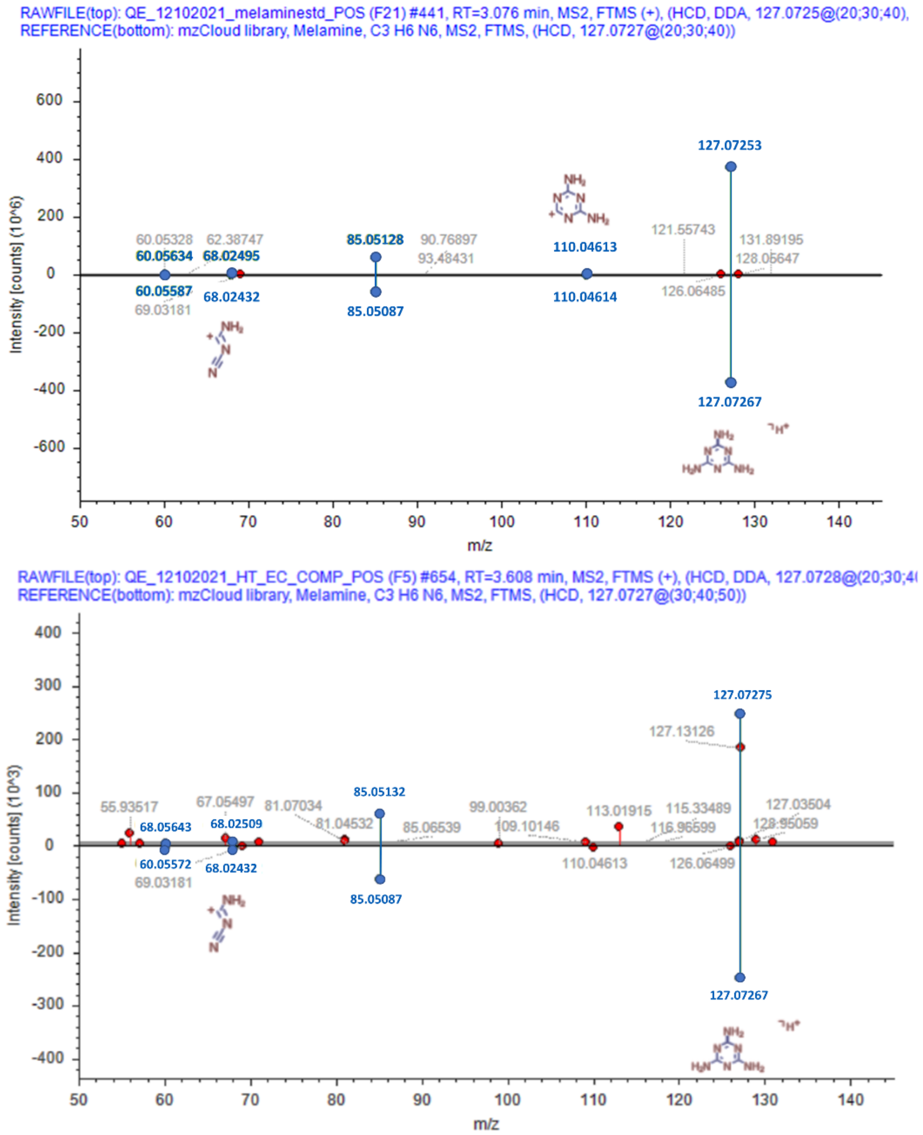

Figure S5. mzCloud database match for melamine standard (top) and fungal treatment extracellular samples (bottom), with blue dots indicating MS^2 spectral matches and red dots indicating mismatches. The mzCloud database match was a 99.995% match.

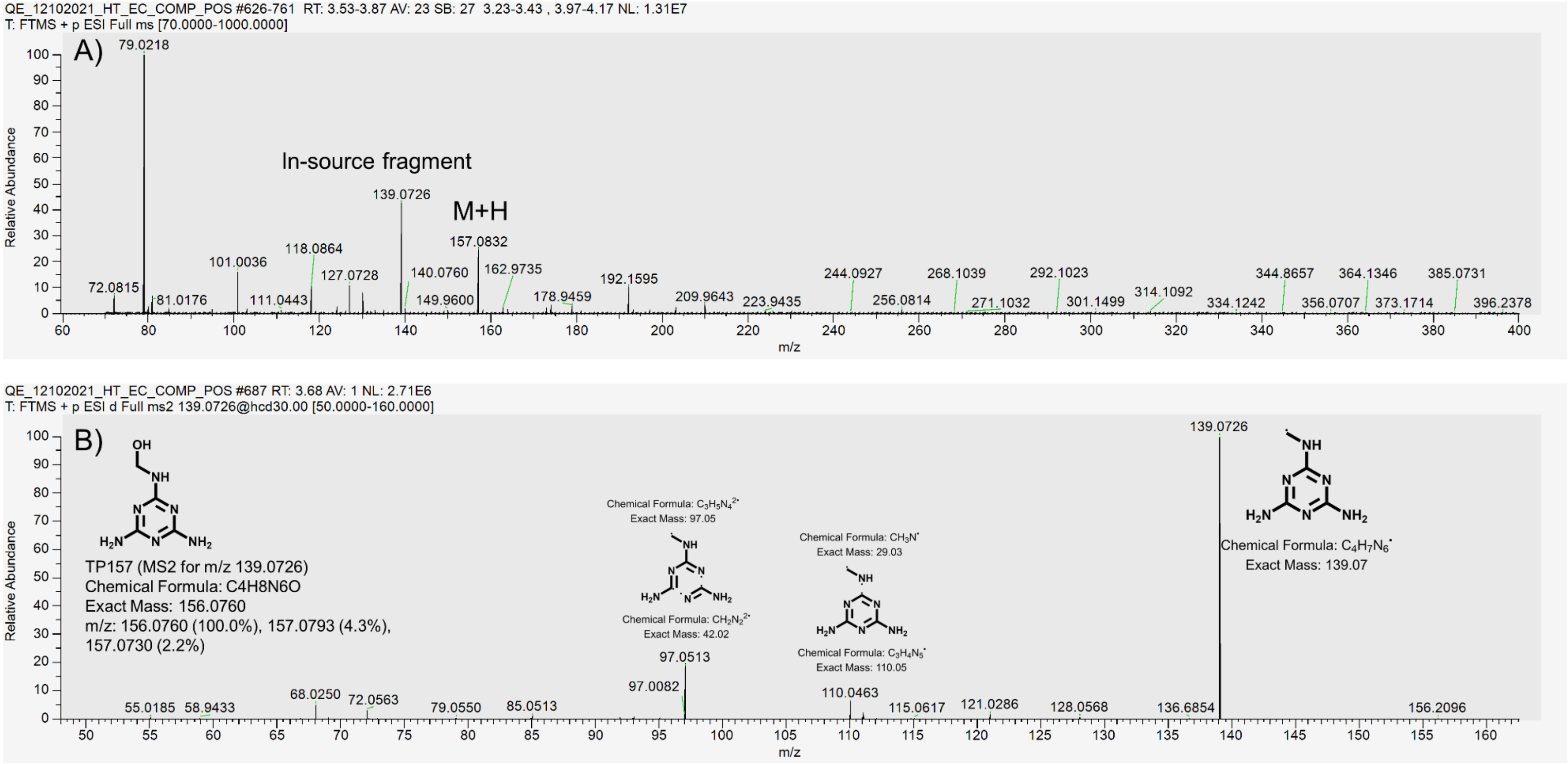

Figure S6. MS[1] (A) and MS[2] (B) spectra for TP157 in extracellular treatment samples

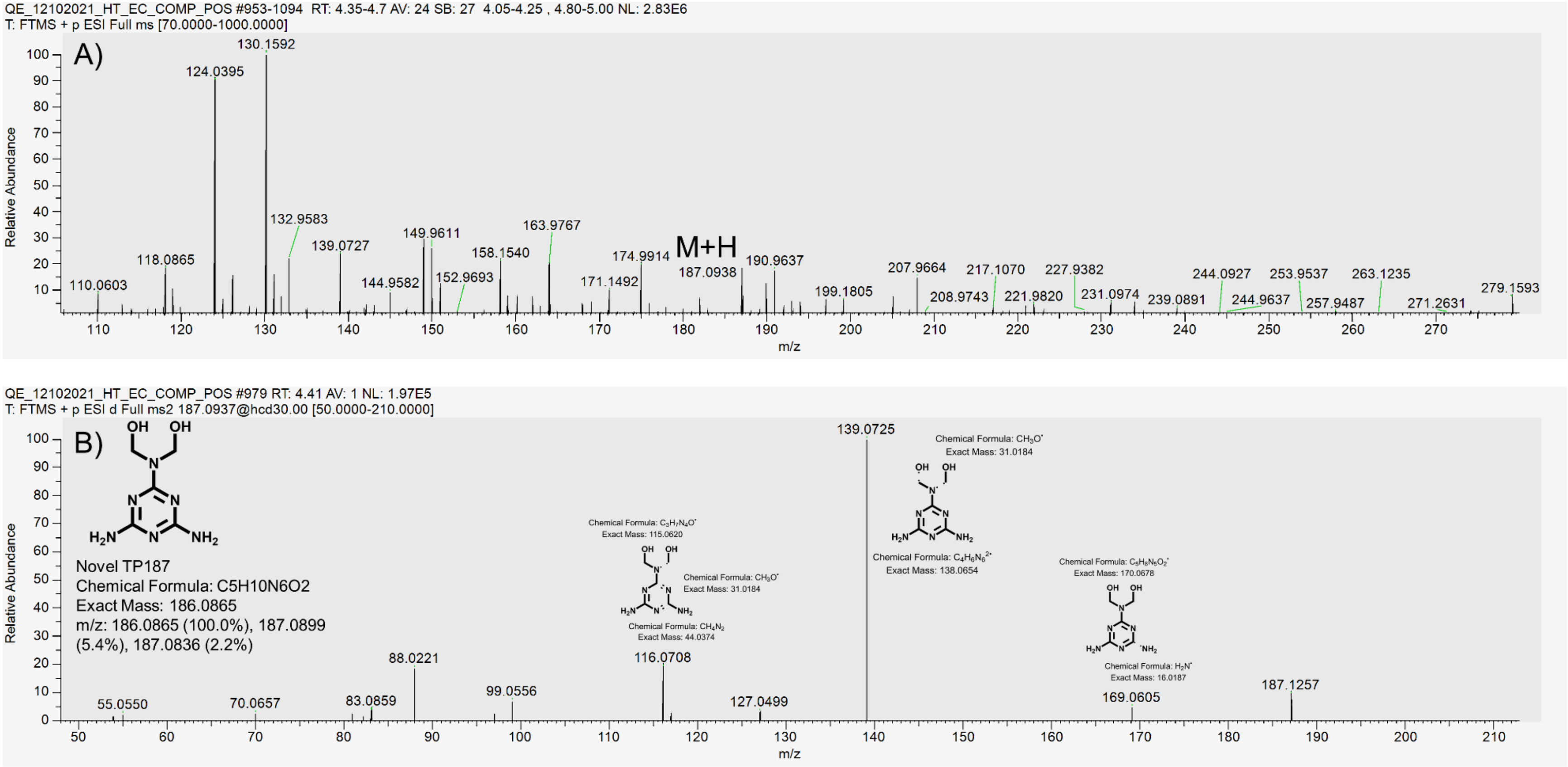

Figure S7. MS[1] (A) and MS[2] (B) spectra for TP187 in extracellular treatment samples.

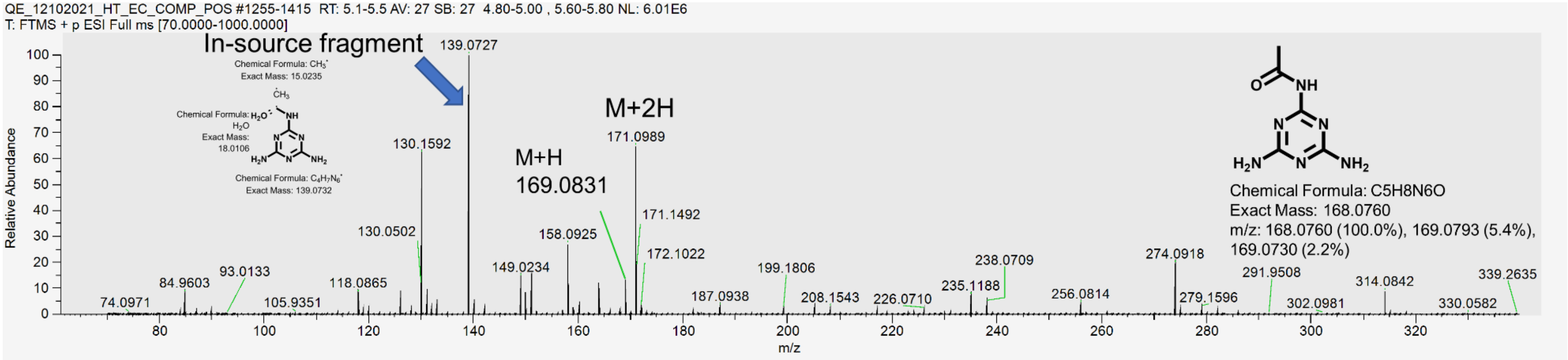

Figure S8. MS[1] spectra for Novel TP169. One in-source fragment from Compound Discoverer at this time point is m/z=139.0727 (C4H6N6). The MS[2] available at this time point is for m/z=151.0726, one of the characteristic fragments when the triaminotriazine ring is preserved. Though no MS[2] is available for the base peak (likely due to small peak intensity), we propose a possible carboxylate structure supported by the chemical formula to a Level 3b confidence.

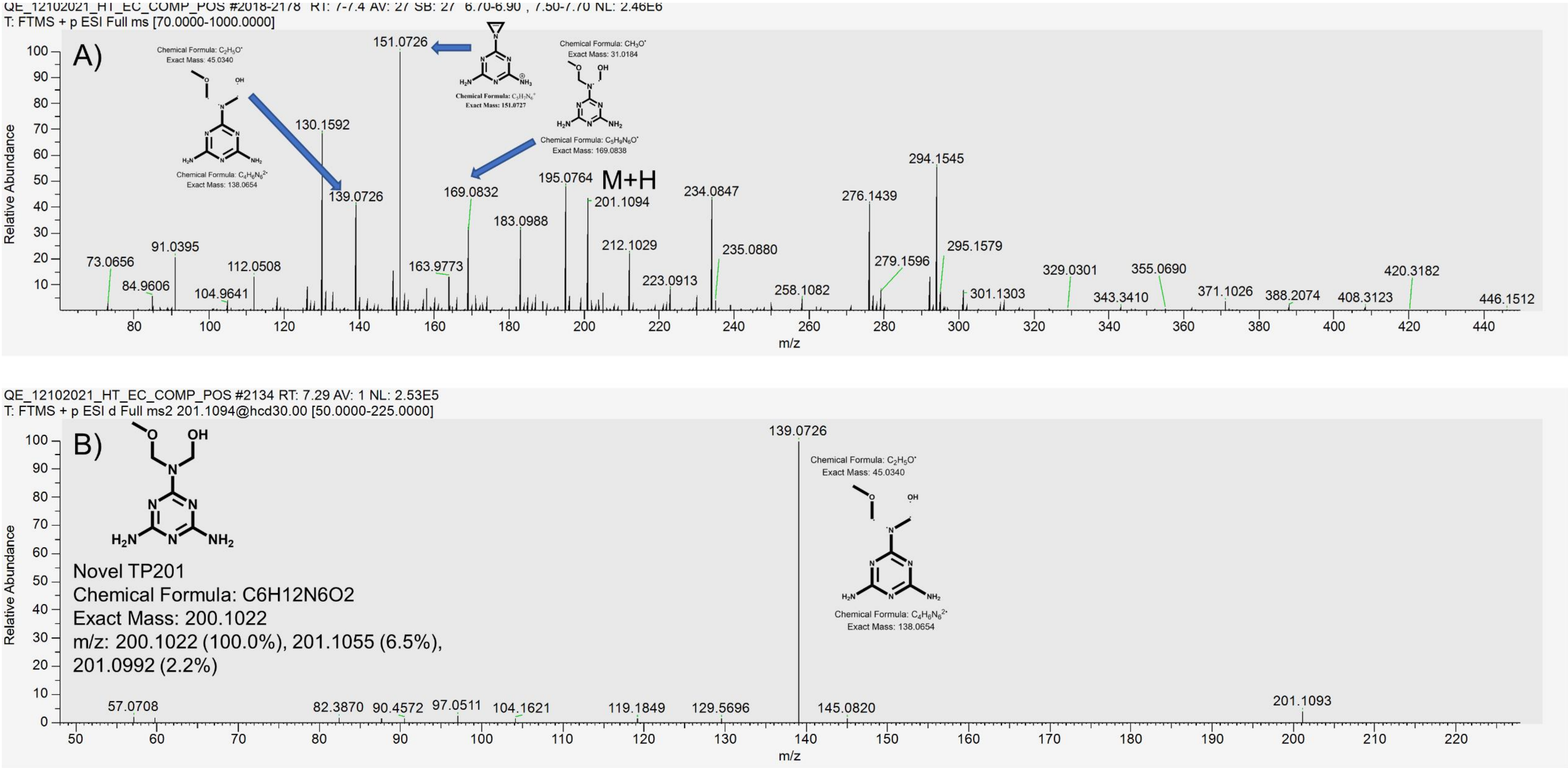

Figure S9. MS[1] (A) and MS[2] (B) spectra for novel TP201 in fungal treatment extracellular samples. In-source fragments detected in Compound Discoverer are labeled in the MS[1] spectra.

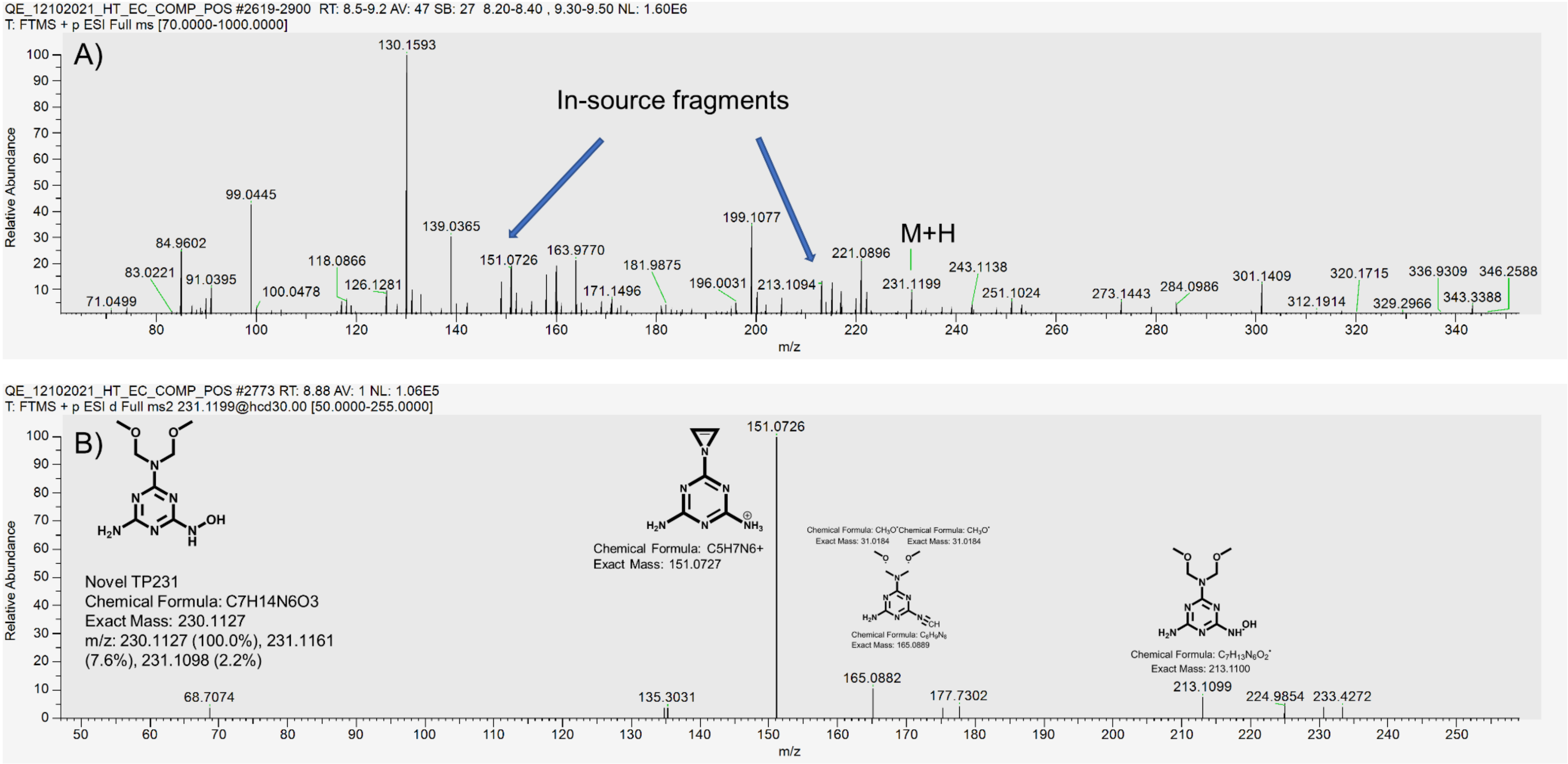

Figure S10. MS[1] (A) and MS[2] (B) spectra for TP231 in fungal treatment extracellular samples. In-source fragments detected in Compound Discoverer are labeled in the MS[1] spectra.

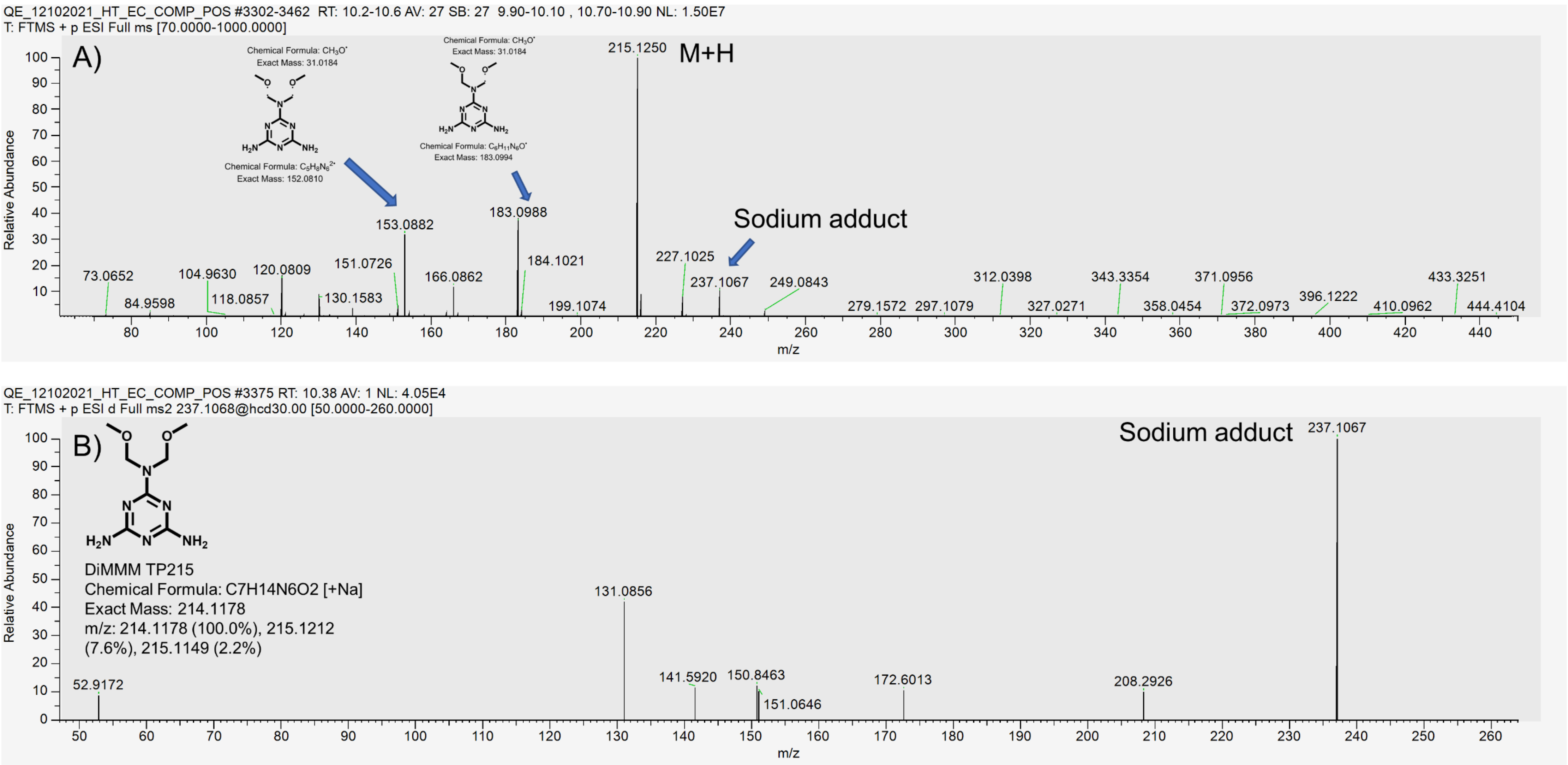

Figure S11. MS[1] (A) and MS[2] (B) spectra for TP215 in fungal treatment extracellular samples. In-source fragments and sodium adducts detected in Compound Discoverer are labeled in the MS[1] spectra. There was no MS[2] spectrum for the m/z=215.1250 feature and observed fragments did not align with Alhelou et al's study (m/z's: 201.1109, 139.0735). Nonetheless, based on unequivocal formula and assignable in-source fragments, we assign this product with a Level 3a confidence.

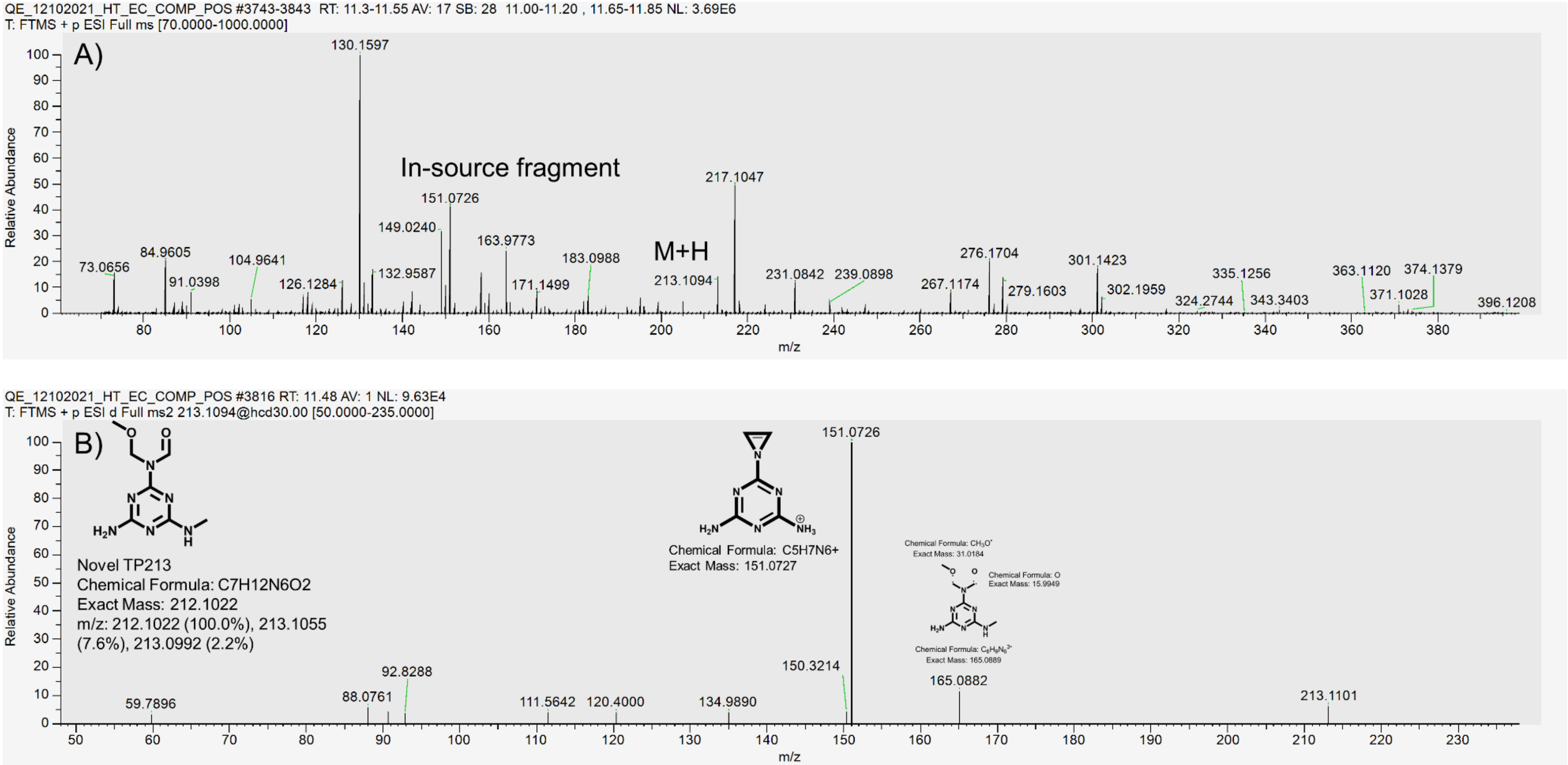

Figure S12. MS^1 (A) and MS^2 (B) spectra for novel TP213 in fungal treatment extracellular samples.

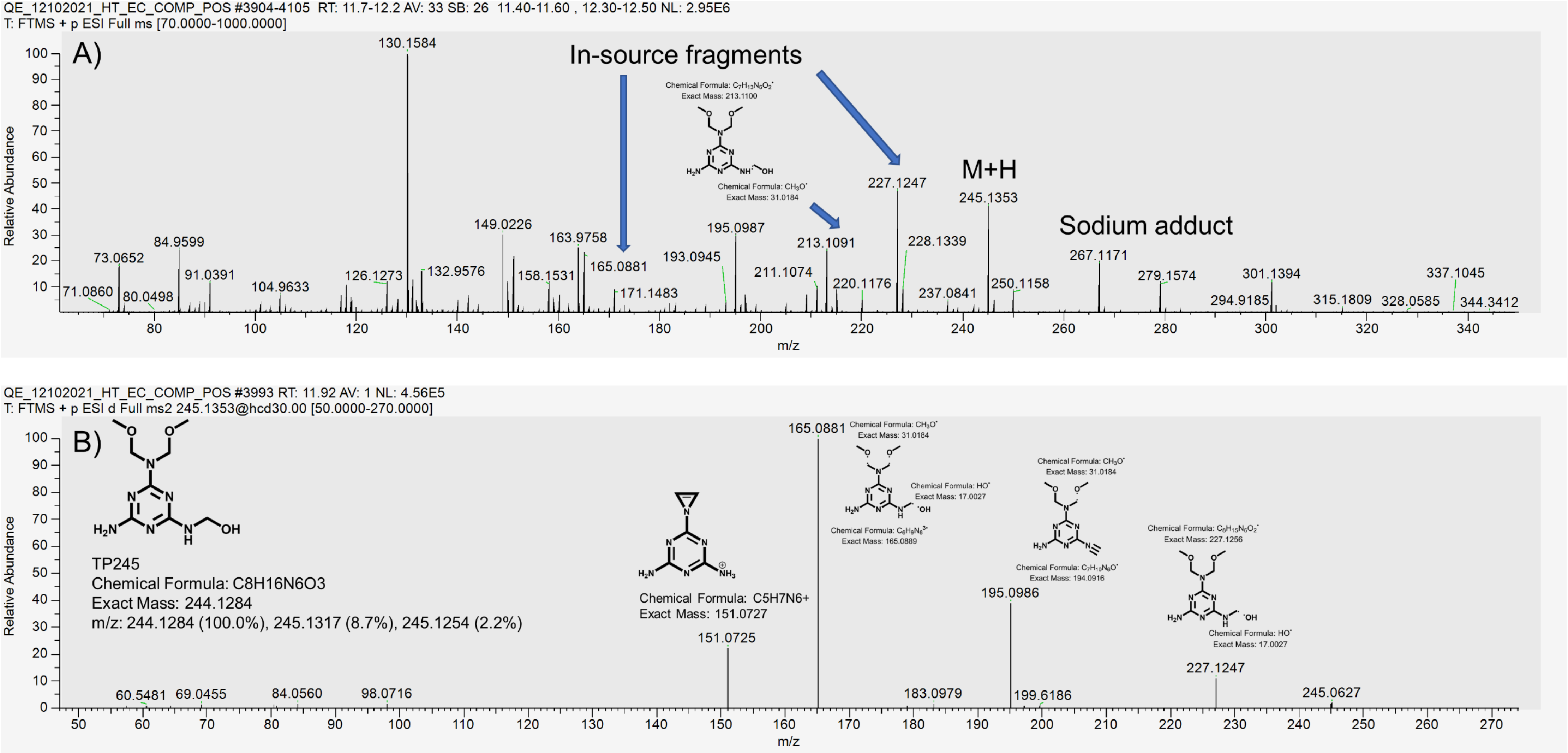

Figure S13. MS[1] (A) and MS[2] (B) spectra for TP245 in fungal treatment extracellular samples. Two fragments (m/z=227.1247 and 195.0986) did not match fragments from Alhelou et al, which led us to identify this product at a Level 3a confidence.

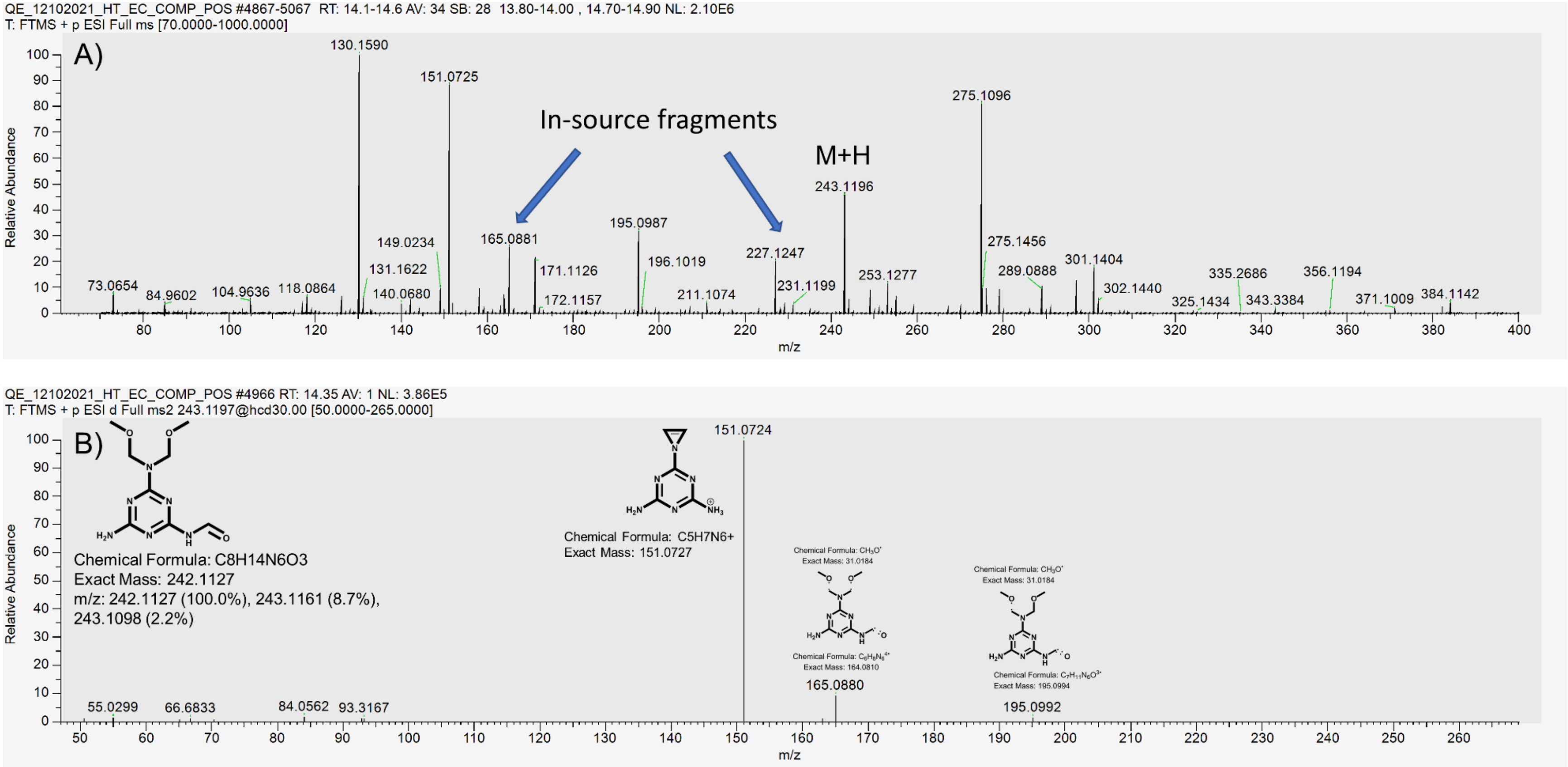

Figure S14. MS[1] (A) and MS[2] (B) spectra for TP243 in fungal treatment extracellular samples. Fragments do not match Alhelou et al., which led us to identify this compound at Level 3a confidence.

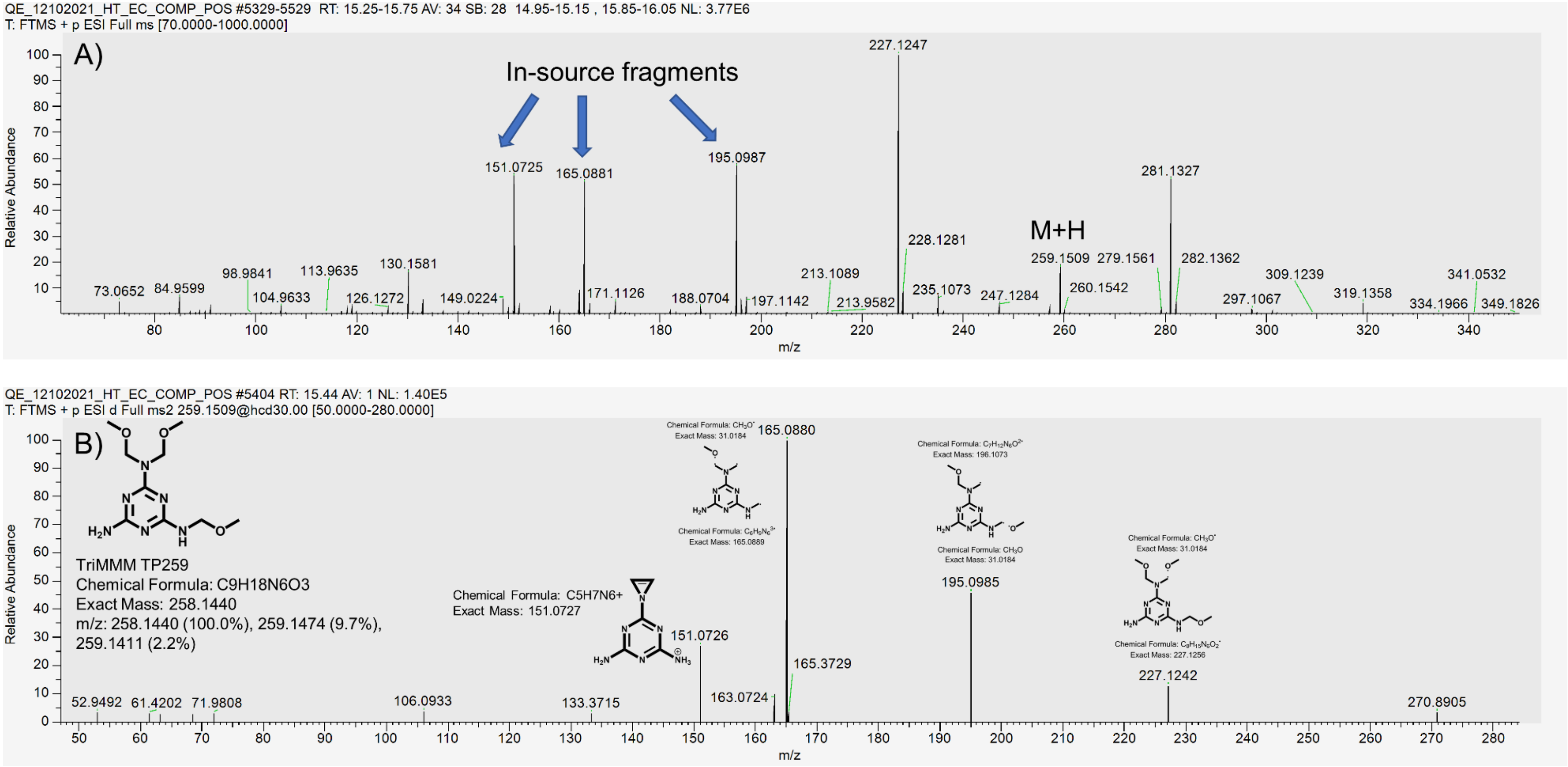

Figure S15. MS[1] (A) and MS[2] (B) spectra for TriMMM (TP259) in fungal treatment extracellular samples. Four of five of the most abundant MS[2] features matched fragments from the Alhelou study, which led us to identify this product with Level 2a confidence.

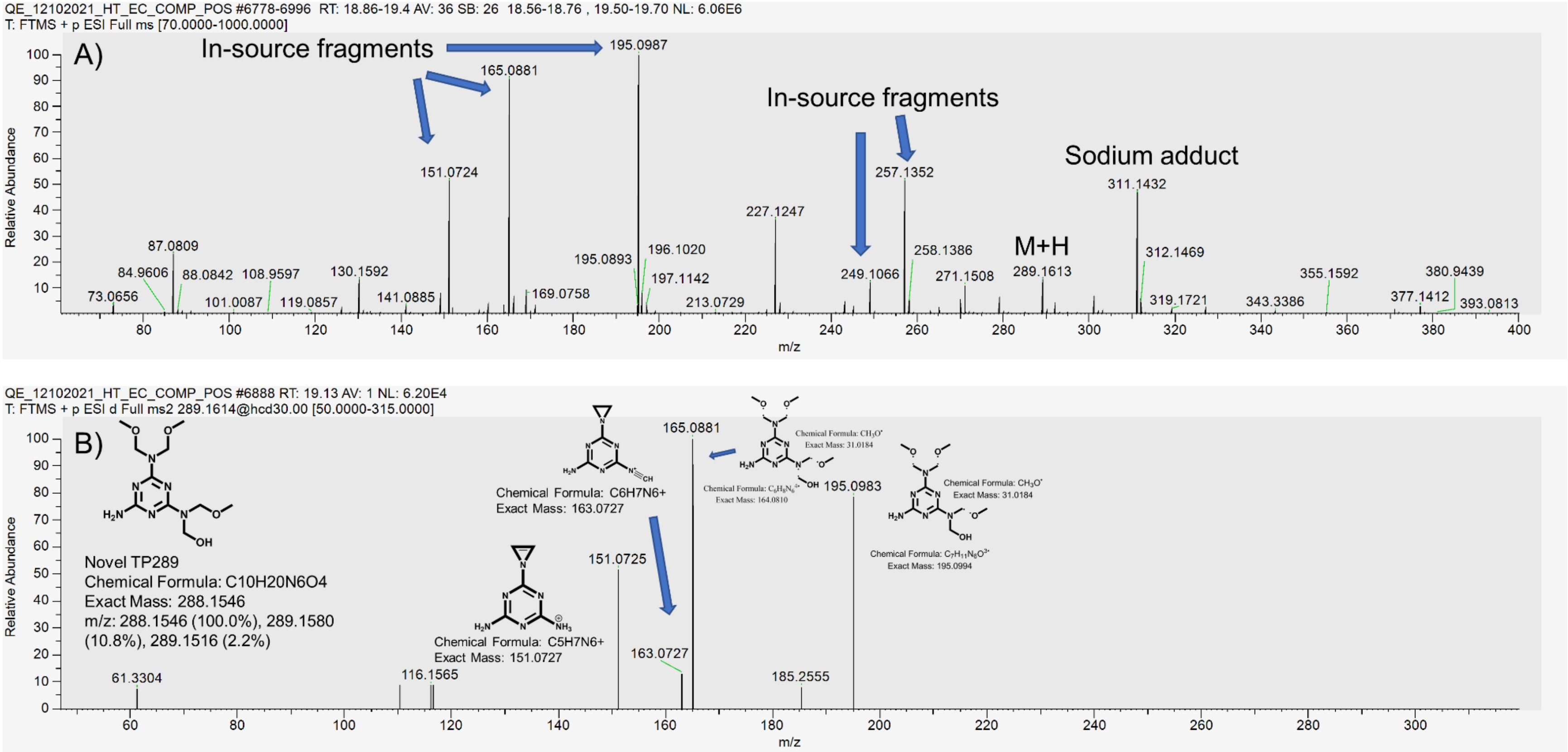

Figure S16. MS[1] (A) and MS[2] (B) spectra for Novel TP289 in fungal treatment extracellular samples. The sodium adduct form was present in Compound Discoverer instead of the parent peak due to signal intensity, which led us to identify this product at a Level 3a confidence.

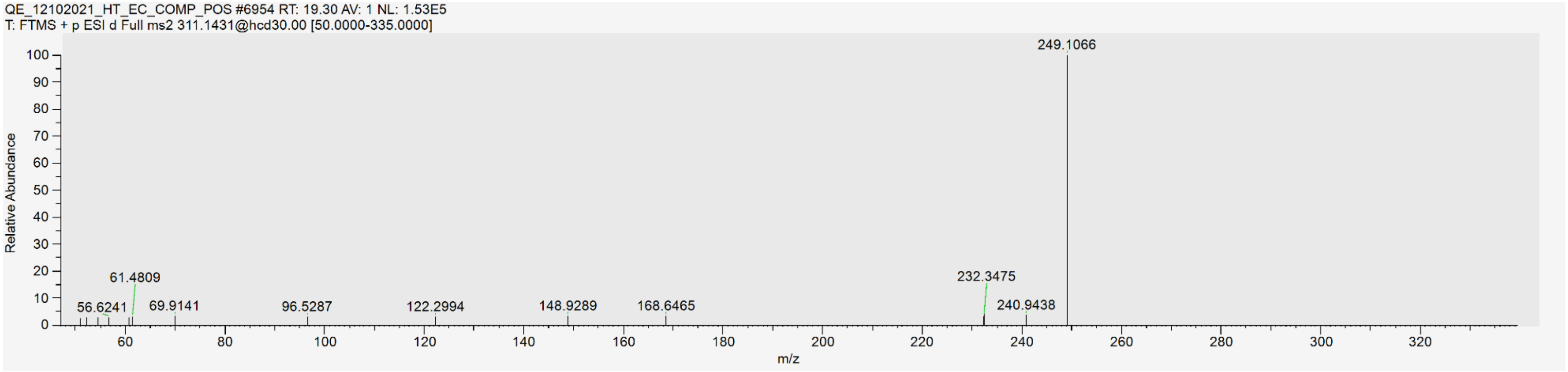

Figure S17. MS[2] spectra for proposed sodium adduct for Novel TP289.

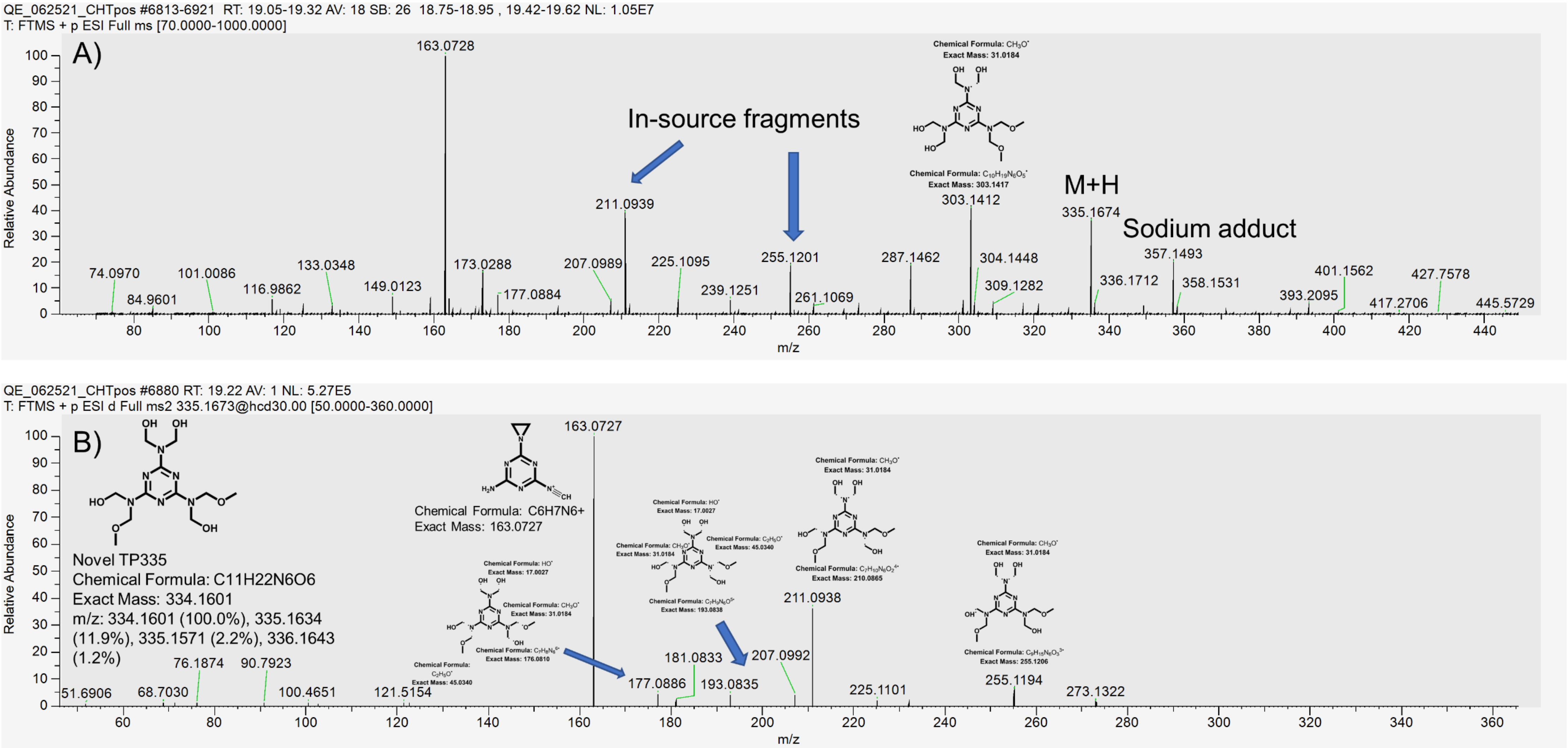

Figure S18. MS[1] (A) and MS[2] (B) spectra for Novel TP335 in fungal treatment biomass-extracted samples. The sodium adduct form was present in Compound Discoverer instead of the parent peak due to signal intensity, which led us to identify this product at a Level 3a confidence. This product also appeared in the extracellular samples, but MS[2]'s were only available in biomass-extracted samples.

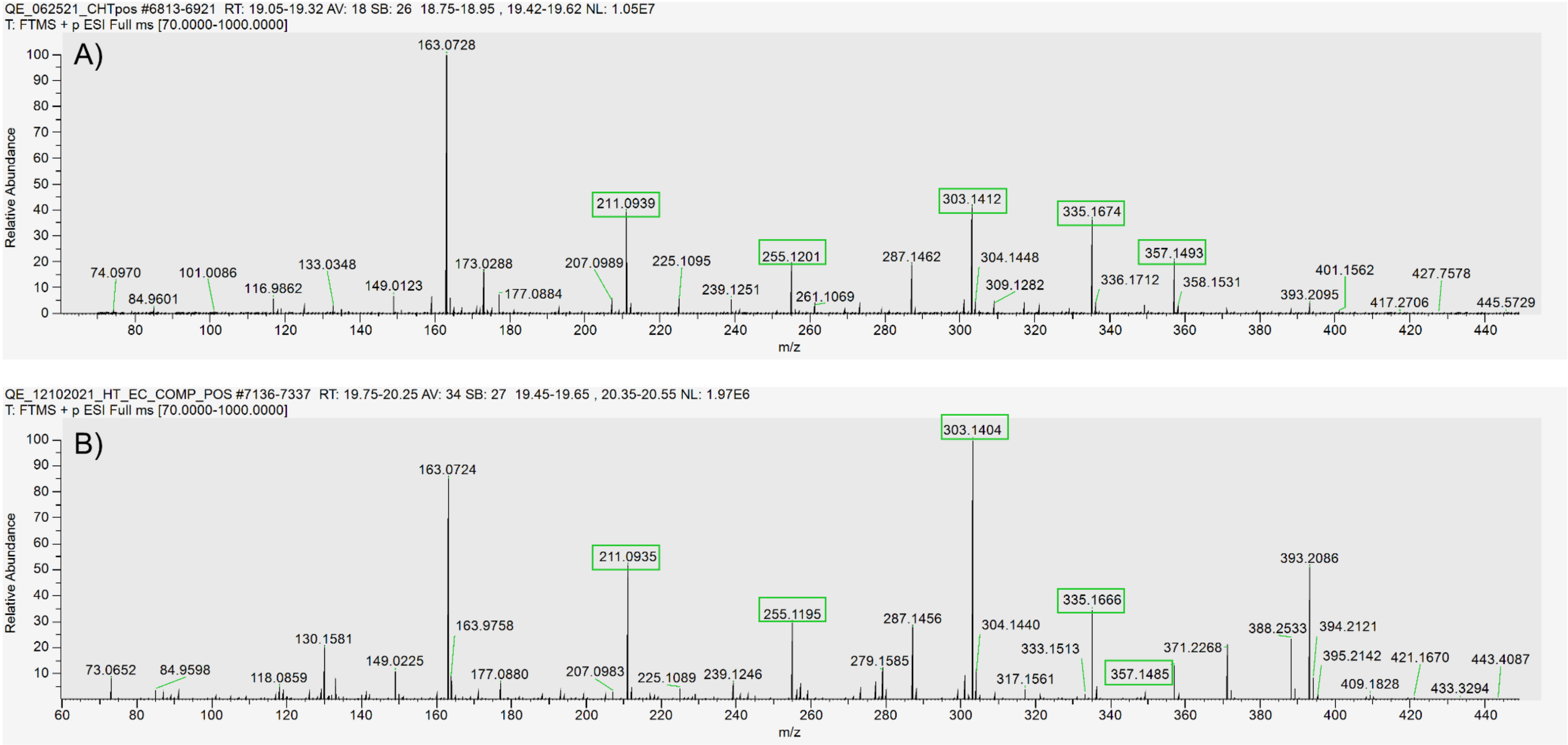

Figure S19. Comparison of MS^1 spectra for biomass-extracted (A) and extracellular (B) samples for the TP335 product. There is a slight retention time difference between these two datasets as they were not analyzed in the same run and a different column (same brand and type) was used (due to column failure). The key matching fragments for this product match and are highlighted with green boxes.

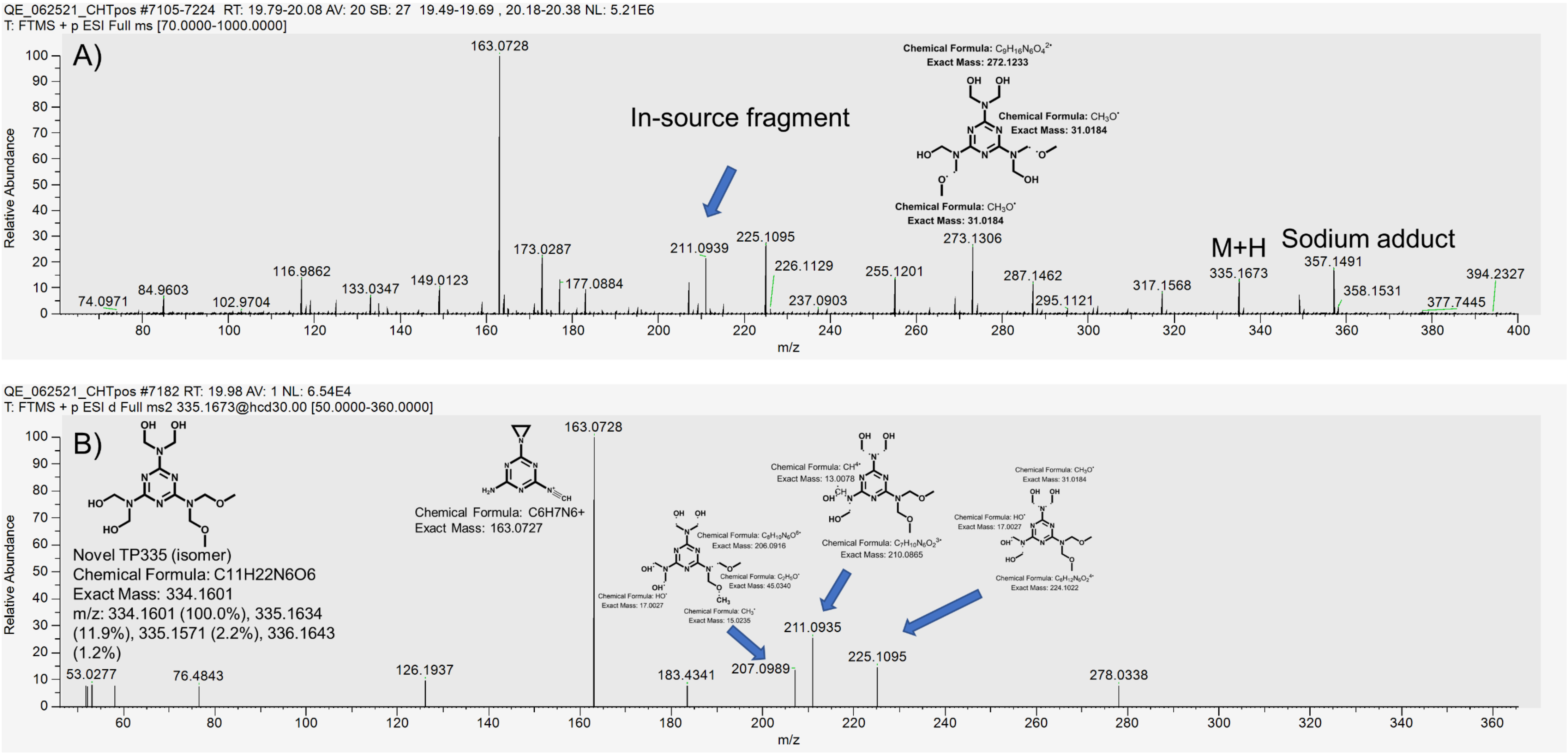

Figure S20. MS[1] (A) and MS[2] (B) spectra for an isomer of Novel TP335 in fungal treatment biomass-extracted samples. We propose an isomer structure due to the similarities in fragmentation and chemical formula but separation in retention time (about one minute). Note that the position of the hydroxyl groups shown here are only one of many possibilities due to symmetry of the product. This product also appeared in the extracellular samples, but MS[2]'s were only available in biomass-extracted samples.

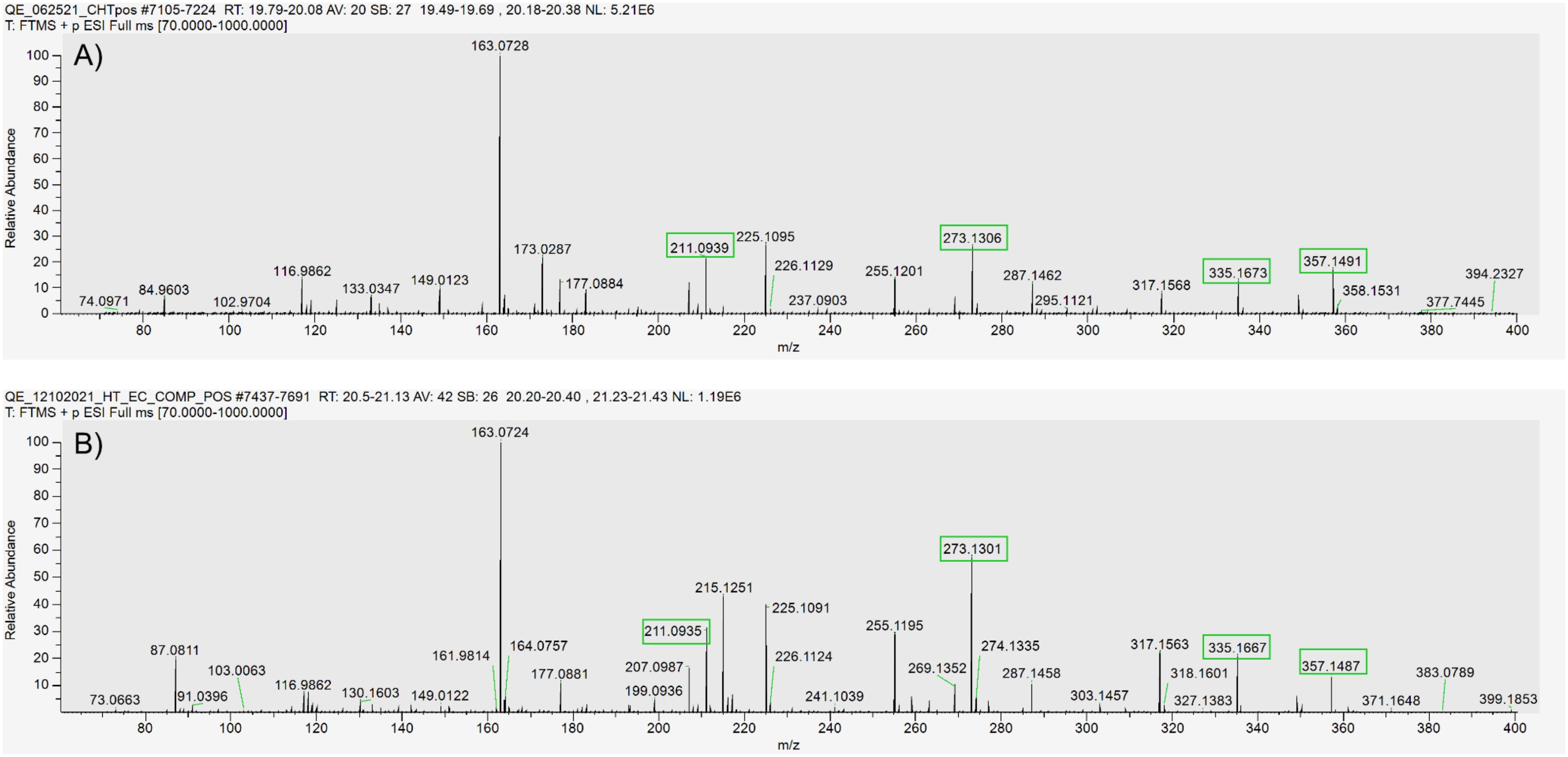

Figure S21. Comparison of MS[1] spectra for biomass-extracted (A) and extracellular (B) samples for the isomer of the TP335 product. There is a slight retention time difference between these two datasets as they were not analyzed in the same run and a different column (same brand and type) was used. The key matching fragments for this product match and are highlighted with green boxes.

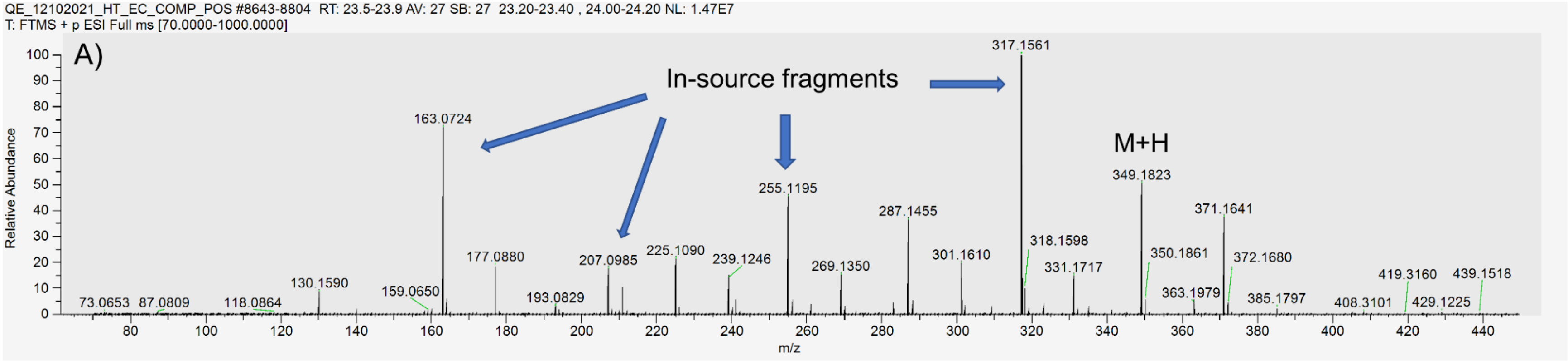

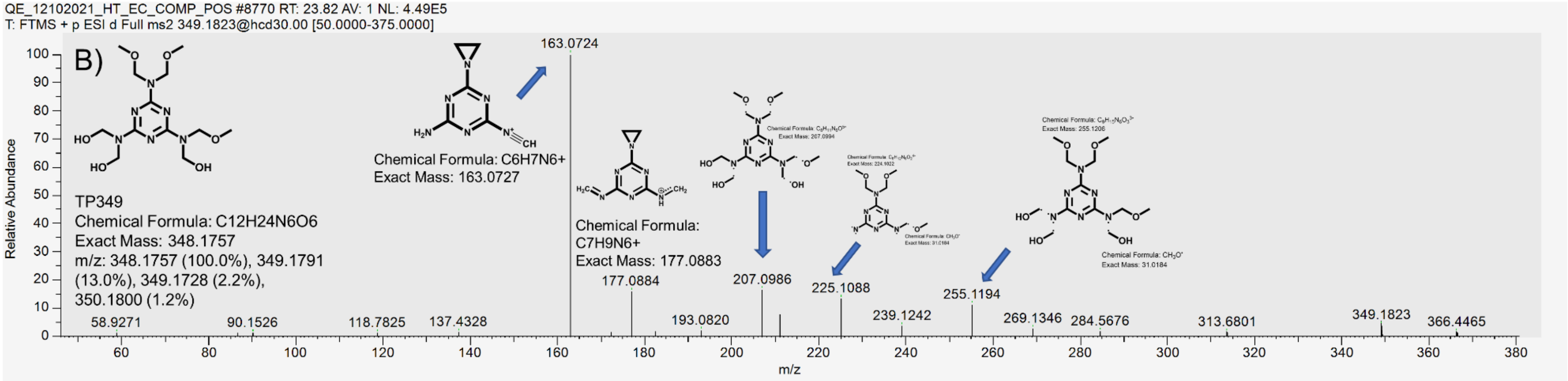

Figure S22. MS[1] (A) and MS[2] (B) spectra for TP349 in fungal treatment extracellular samples. Only the m/z=163.0731 and the sodium adduct at m/z=371.164 match the Alhelou et al. study, but the ability to propose top 5 abundant fragments that are consistent with the structure of TP349 led us to identify this product at Level 2b confidence.

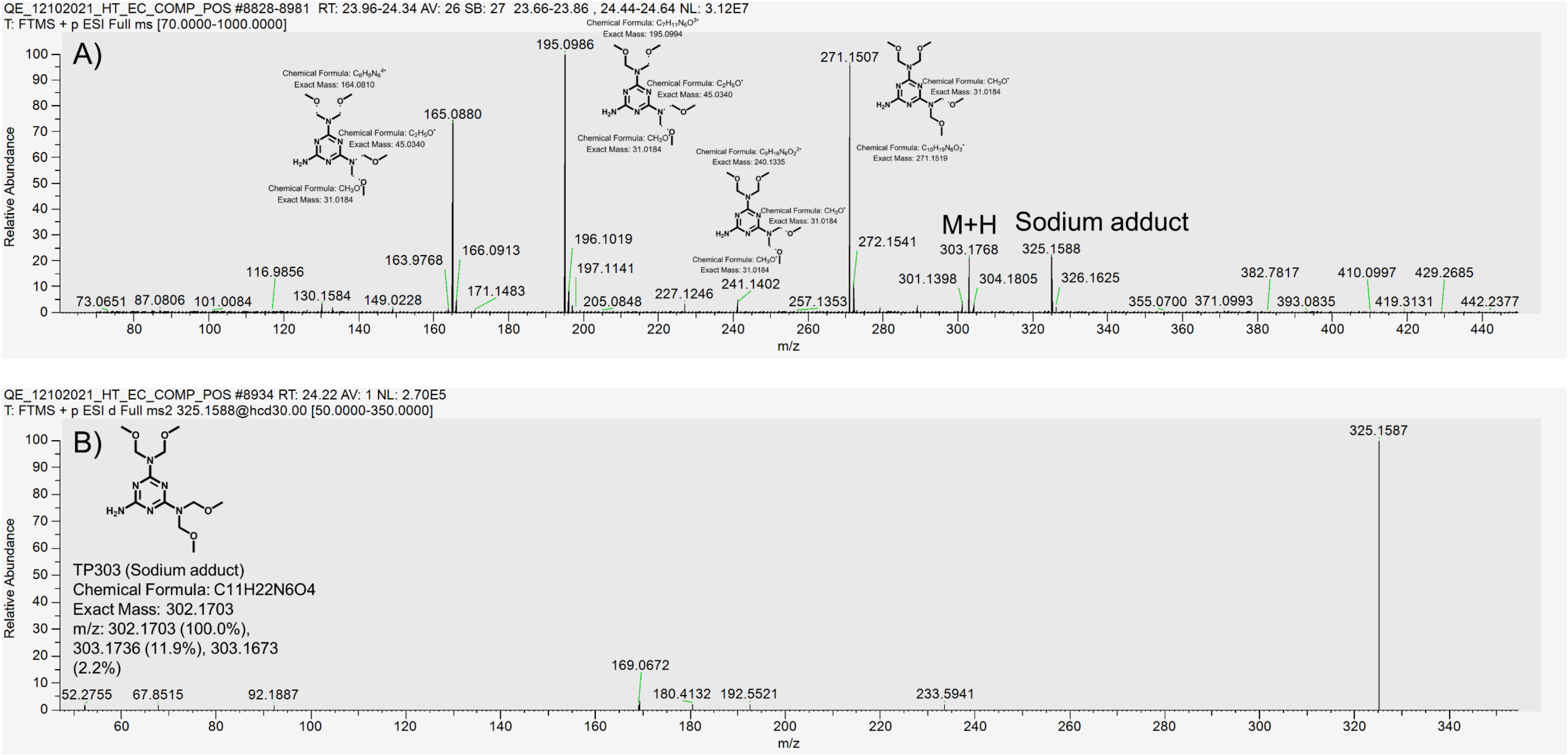

Figure S23. MS[1] (A) and MS[2] (B) spectra for TP303 in fungal treatment extracellular samples. Though no MS[2] exists for the parent TP303, our in-source fragments detected in the MS[1] are consistent with the fragmentation pattern in Alhelou et al. The following m/z in the MS[1] match: 325.1588 (sodium adduct), 303.1768 (M+H), 271.1507, 241.1402, 195.0986, 165.0880.

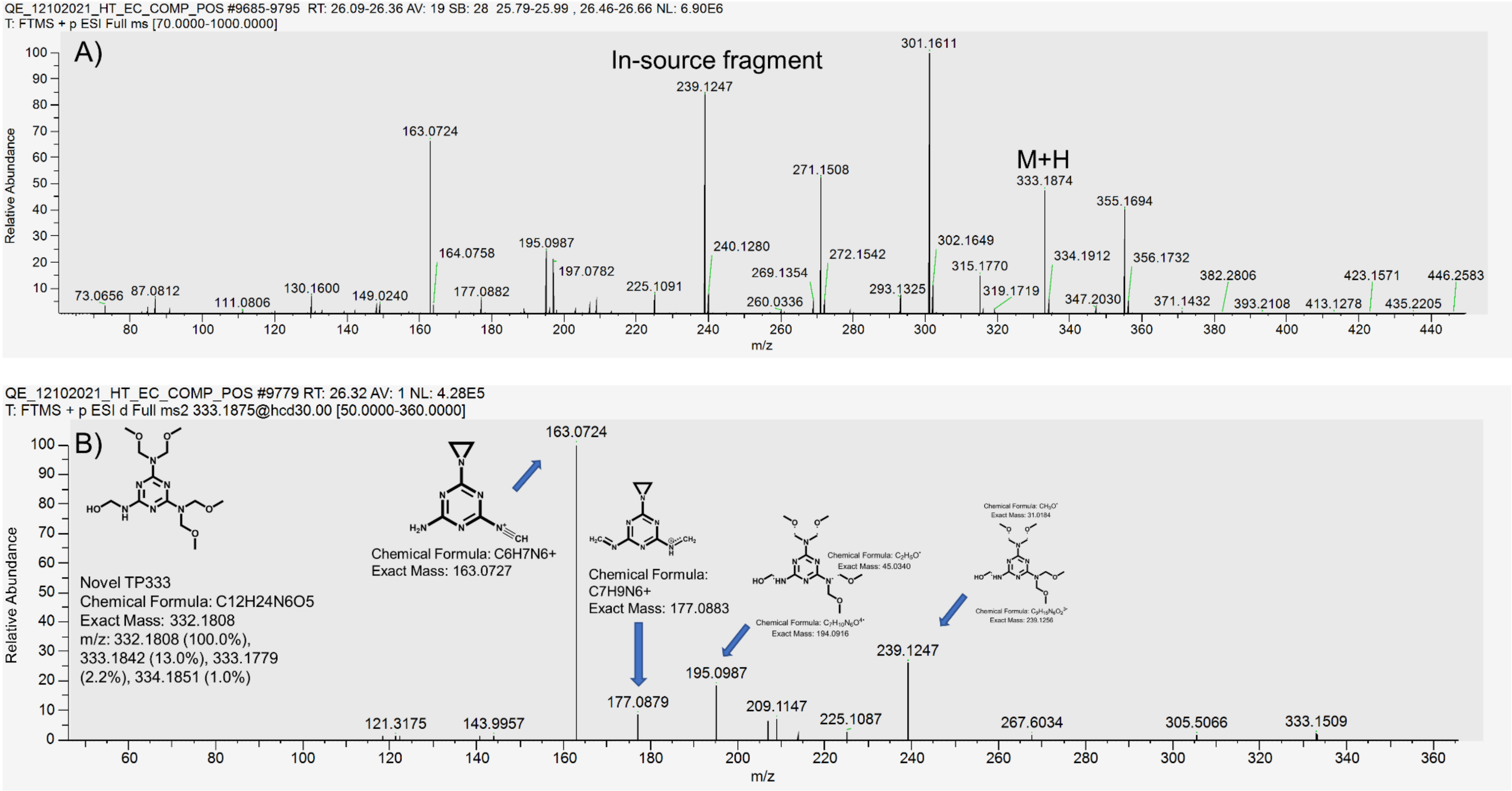

Figure S24. MS^1 (A) and MS^2 (B) spectra for novel TP333 in fungal treatment extracellular samples.

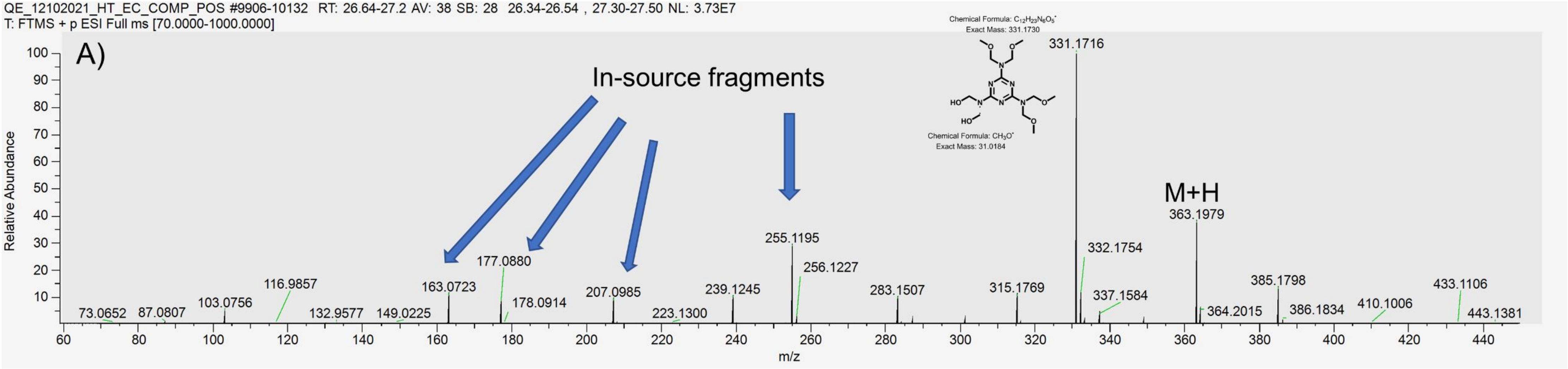

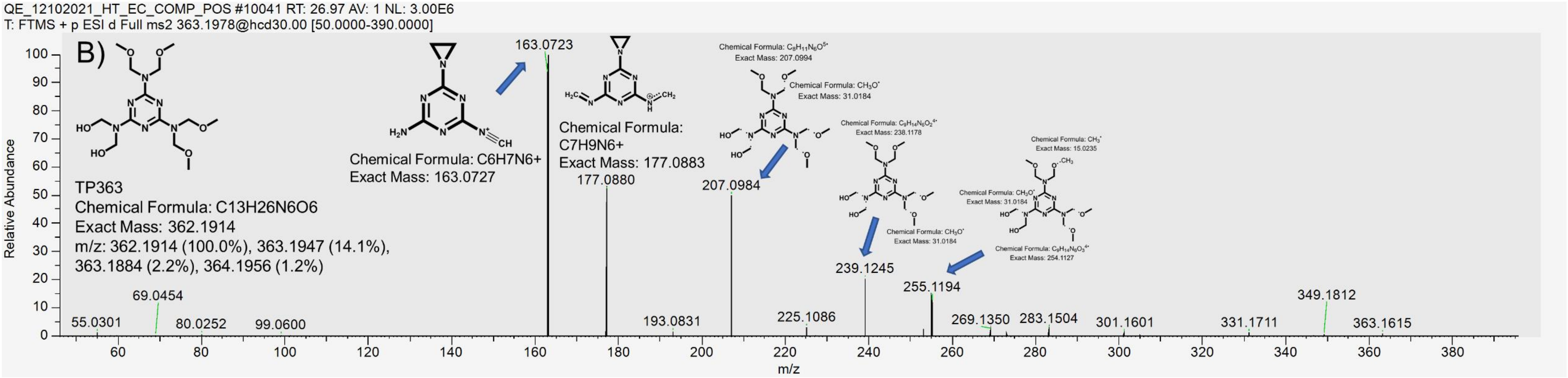

Figure S25. MS[1] (A) and MS[2] (B) spectra for TP363 in fungal treatment extracellular samples. The following in-source fragments from the MS[1] or fragments from the MS[2] match Alhelou et al: 331.1716, 283.1504, 255.1194, 239.1245, 207.0984, 177.0883, 163.0727. All of the 5 most abundant features in the MS[2] match fragments from Alhelou et al (intensity/relative intensity not reported in that study.)

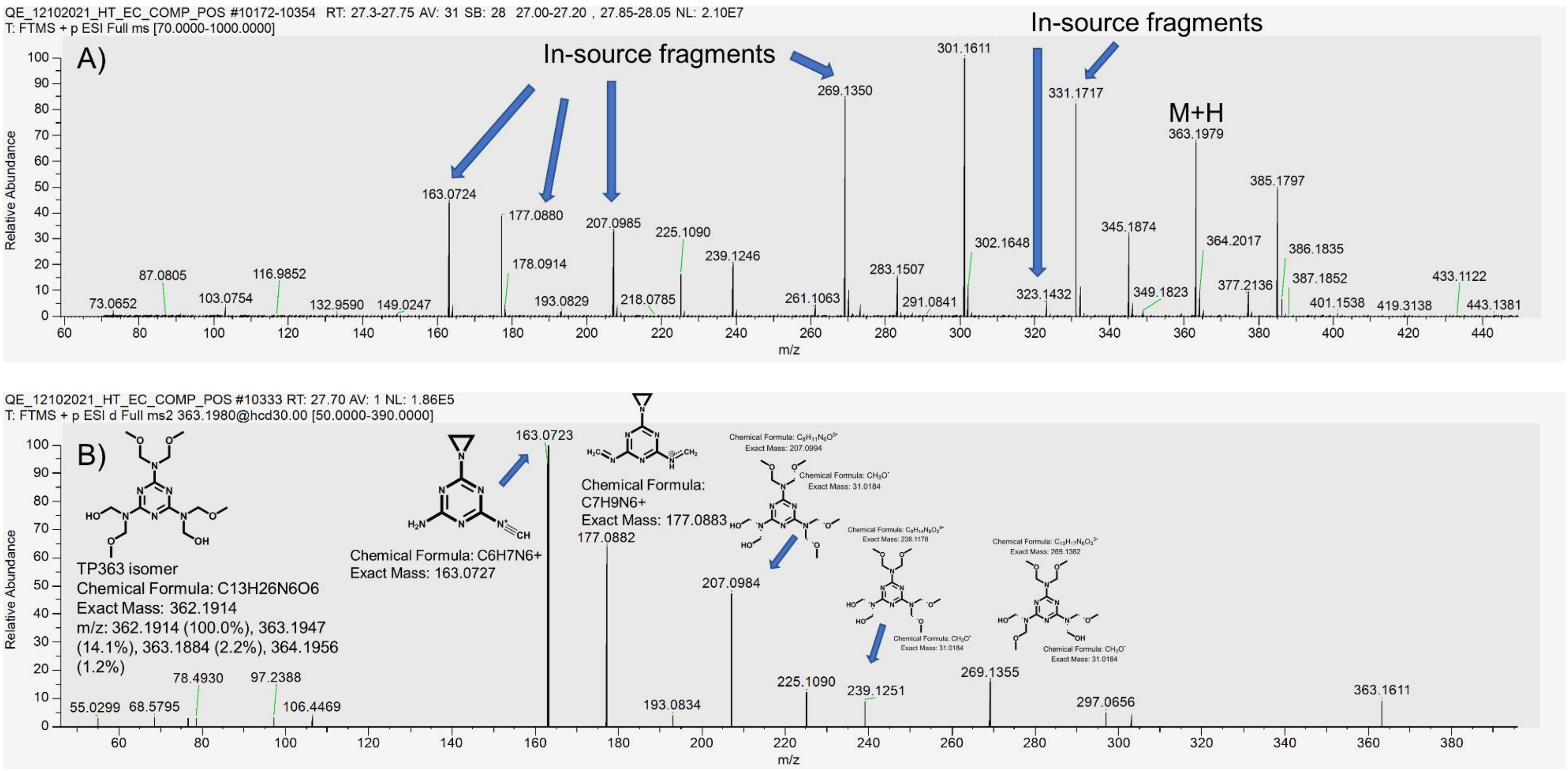

Figure S26. MS[1] (A) and MS[2] (B) spectra for proposed novel isomer of the TP363 in fungal treatment extracellular samples. The following in-source fragments from the MS[1] or fragments from the MS[2] match Alhelou et al: 331.1717, 283.1507, 239.1251, 207.0984, 177.0882, 163.0727. The product at RT 26.97 was proposed as the structure from Alhelou's study based on more matching fragments, but we caution that the hydroxyl positions cannot be reasonably determined from the spectra; this proposition was made for purpose of consistency across the literature. Given the difference of 0.73 minutes and similar fragmentation, the RT 26.97 and RT 27.7 products were identified as isomers with Level 3a confidence.

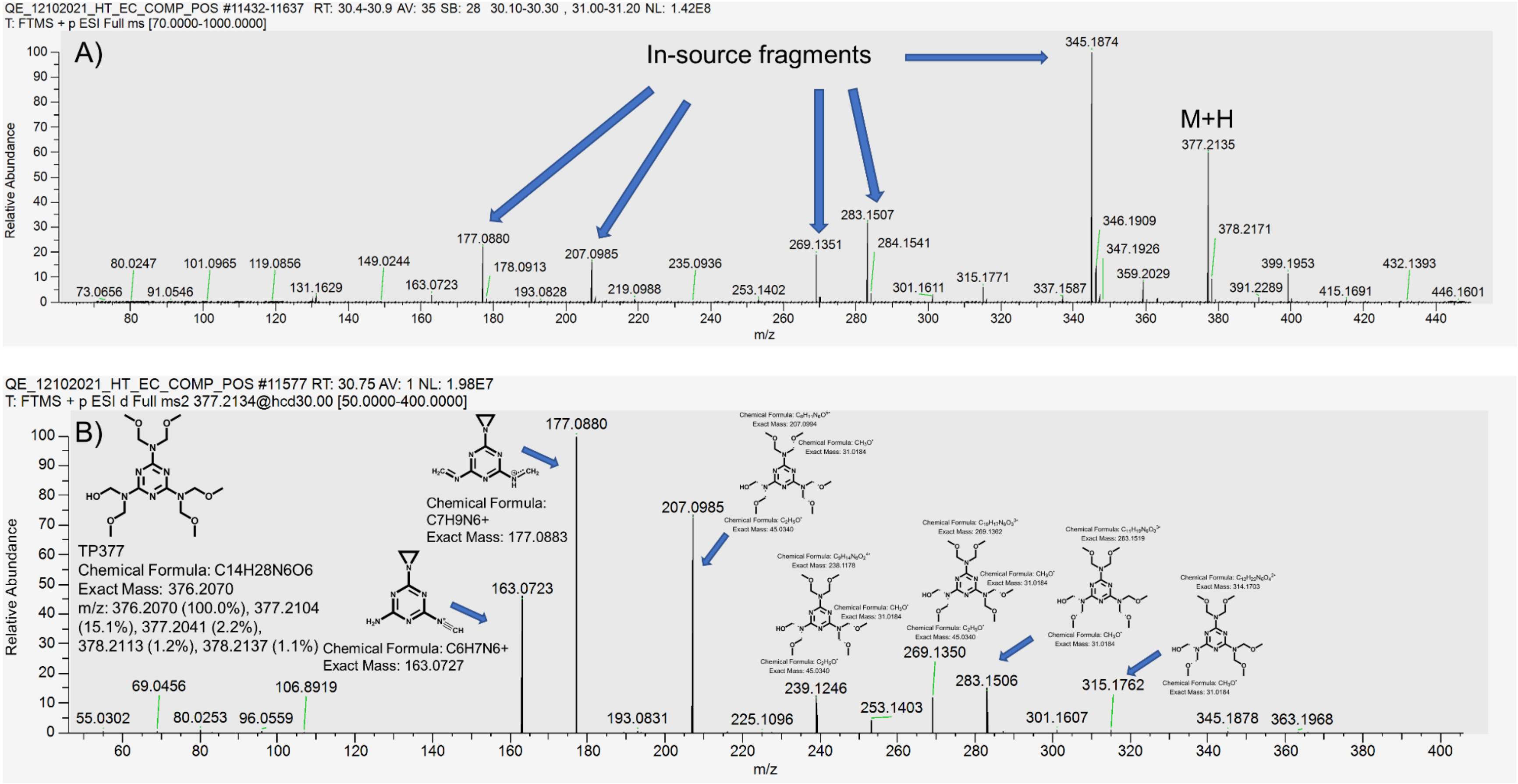

Figure S27. MS[1] (A) and MS[2] (B) spectra for TP377 in fungal treatment extracellular samples. The following in-source fragments from the MS[1] or fragments from the MS[2] match Alhelou et al: 345.1874, 315.1762, 283.1506, 269.1350, 239.1246, 207.0985, 177.0880, 163.0723. Given similarity of fragmentation, we identified this to a Level 2a confidence.

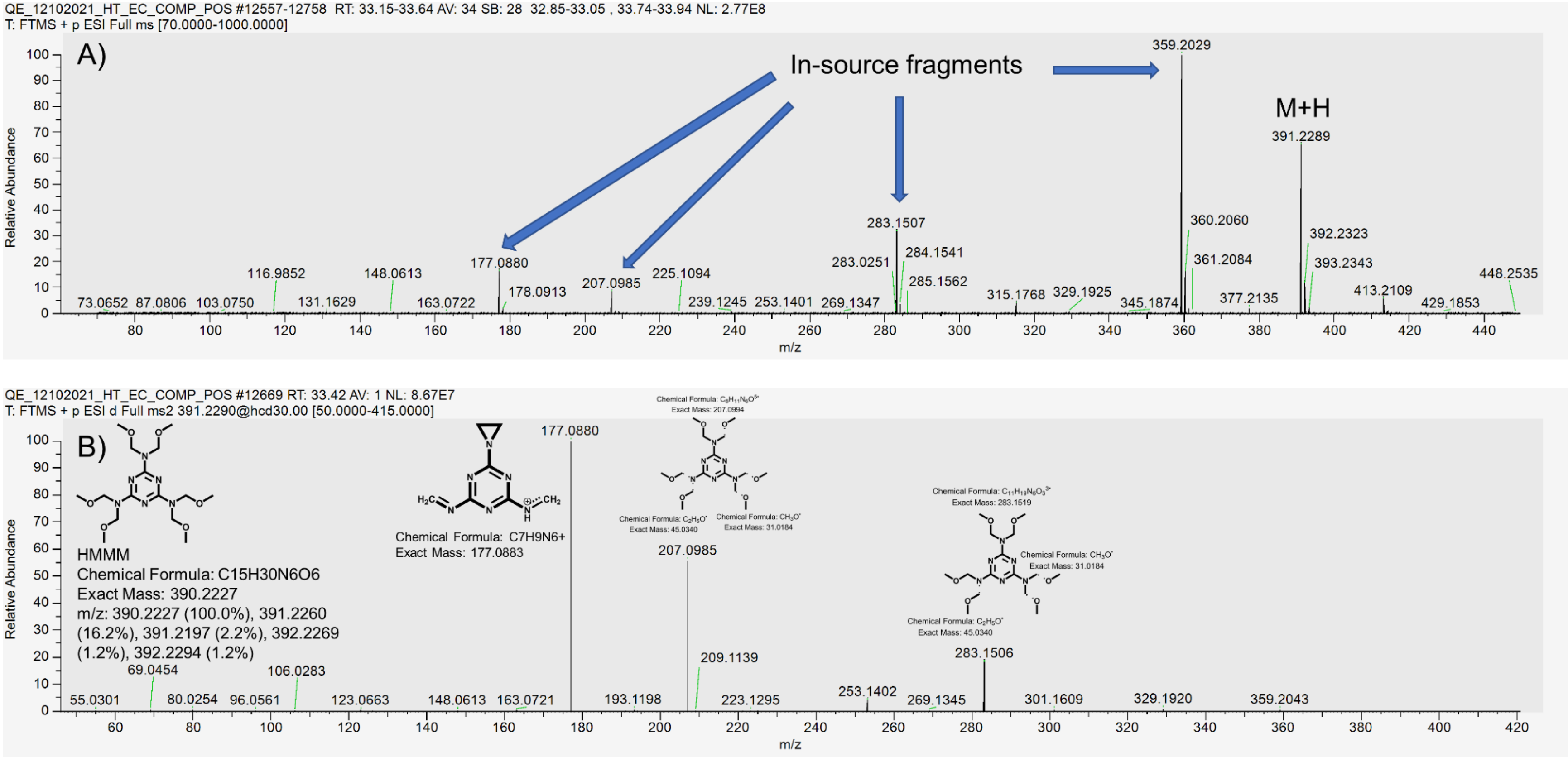

Figure S28. MS[1] (A) and MS[2] (B) spectra for HMMM in fungal treatment extracellular samples. The parent HMMM was identified to a Level 1 confidence using a standard and retention time matching. Additionally, there was a 99.9% similarity with spectra from the mzCloud database (Reference ID 2645).

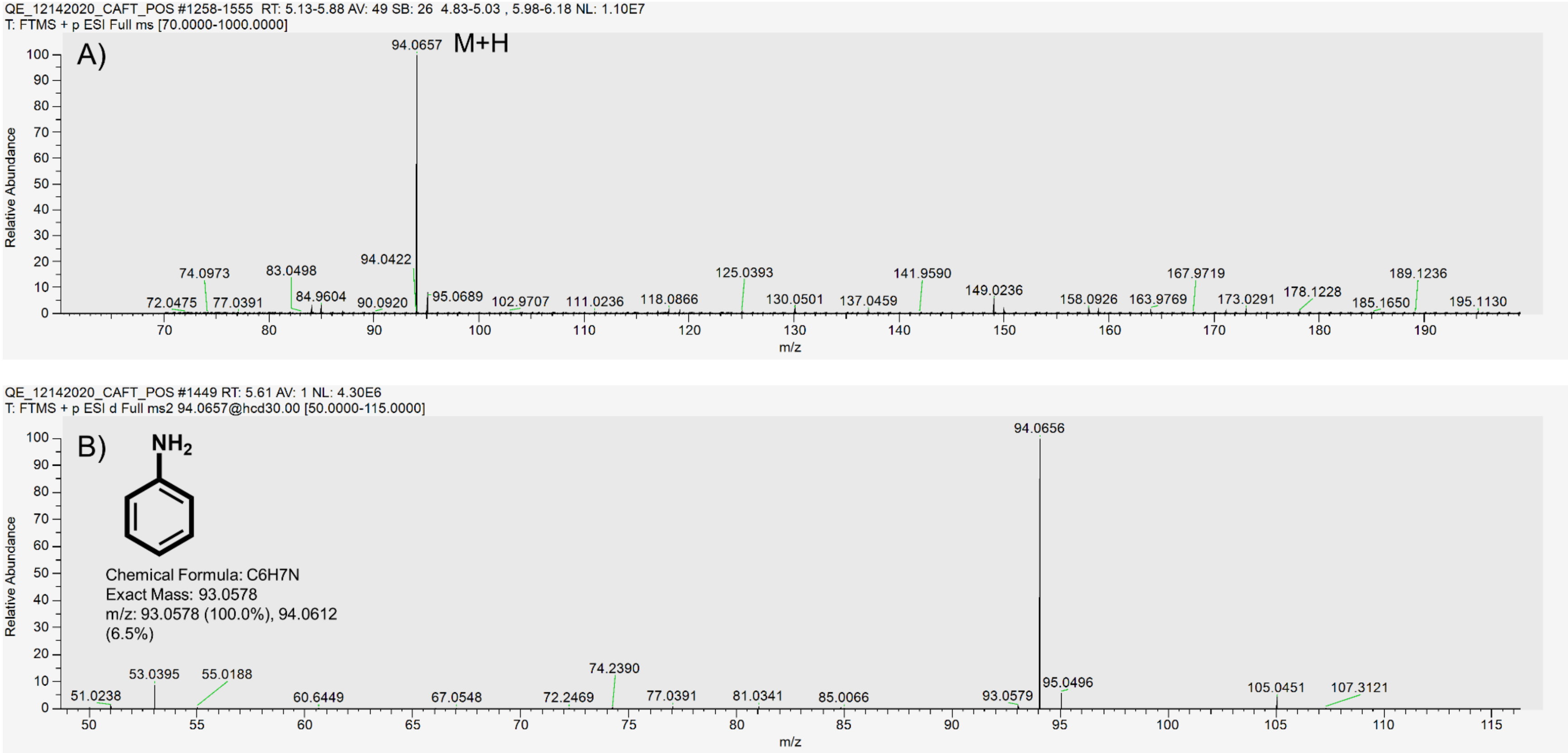

Figure S29. MS[1] (A) and MS[2] (B) spectra for aniline in fungal treatment biomass-extracted samples. There was a 99.1% similarity with spectra from the mzCloud database (Reference ID 823). Given this high similarity and absence of other significantly up-regulated features in the MS[1] at this retention time, we identified this as aniline with a Level 2a confidence.

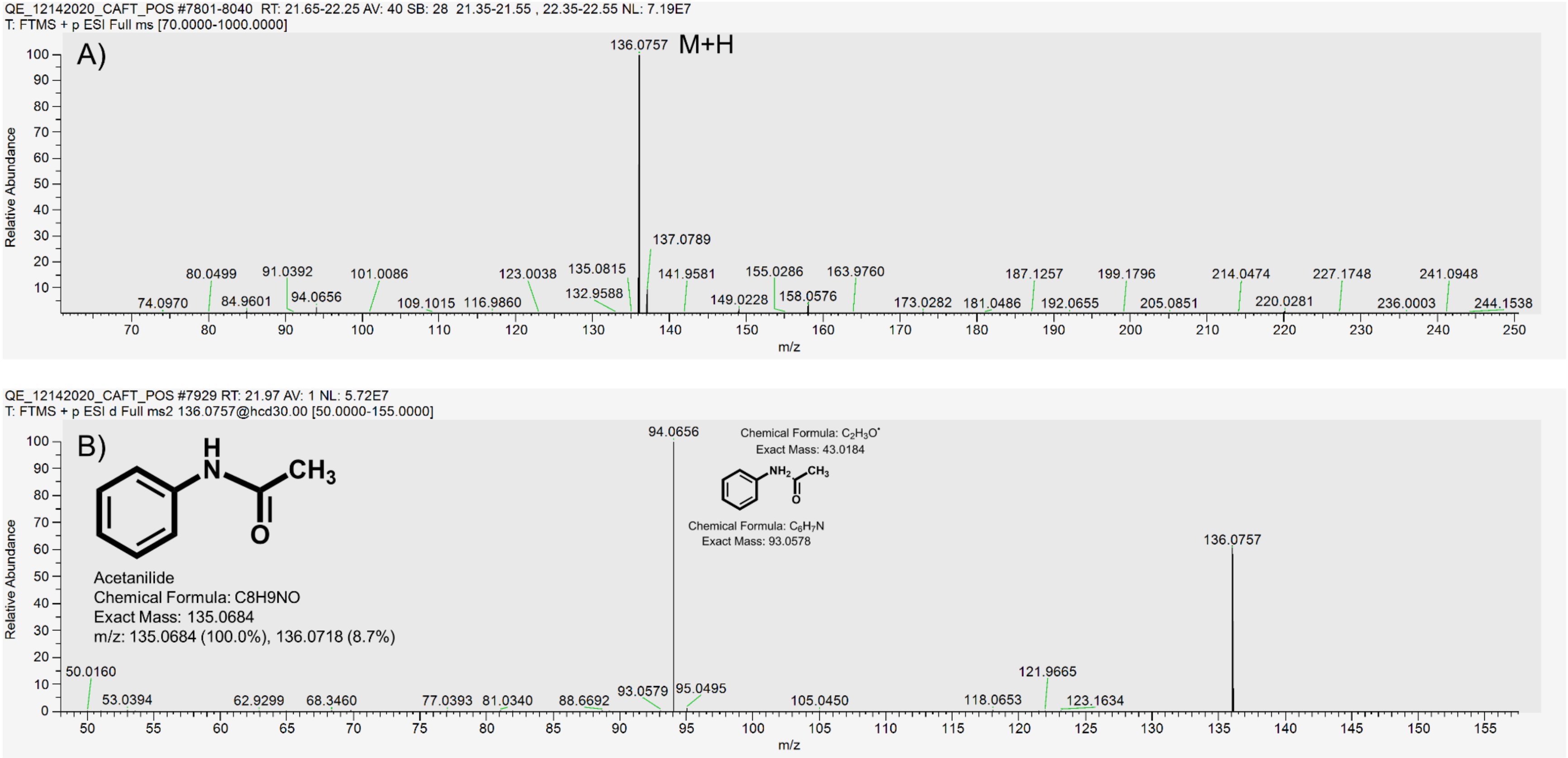

Figure S30. MS[1] (A) and MS[2] (B) spectra for acetanilide in fungal treatment biomass-extracted samples. The parent HMMM was identified to a Level 1 confidence using a standard and retention time matching. Additionally, there was a 99% similarity with spectra from the mzCloud database (Reference ID 637).

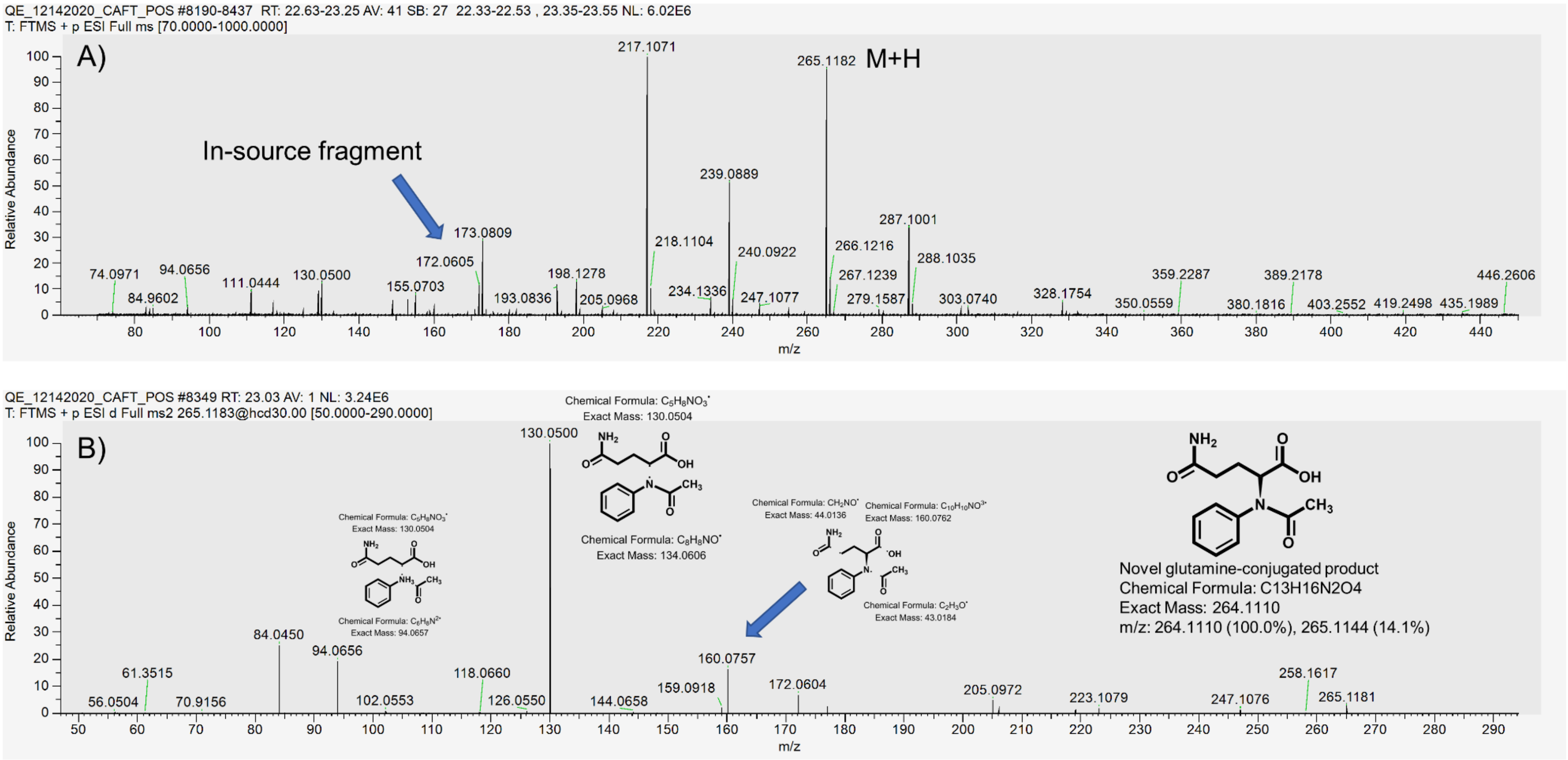

Figure S31. MS[1] (A) and MS[2] (B) spectra for a novel glutamine-conjugated product of acetanilide in fungal treatment biomass-extracted samples. The closest mzCloud match is for N-Phenylacetylglutamine (89%, reference ID 541).
